# A Water-Free In Situ HF Treatment for Ultrabright InP Quantum Dots

**DOI:** 10.1021/acs.chemmater.2c02800

**Published:** 2022-11-04

**Authors:** Reinout F. Ubbink, Guilherme Almeida, Hodayfa Iziyi, Indy du Fossé, Ruud Verkleij, Swapna Ganapathy, Ernst R. H. van Eck, Arjan J. Houtepen

**Affiliations:** † Optoelectronic Materials Section, Faculty of Applied Sciences, Delft University of Technology, Van der Maasweg 9, 2629 HZ Delft, The Netherlands; ‡ Department of Radiation Science and Technology, Faculty of Applied Sciences, Delft University of Technology, 2629 JB Delft, The Netherlands; § Magnetic Resonance Research Center, Institute for Molecules and Materials, Radboud University, 6525 AJ Nijmegen, The Netherlands

## Abstract

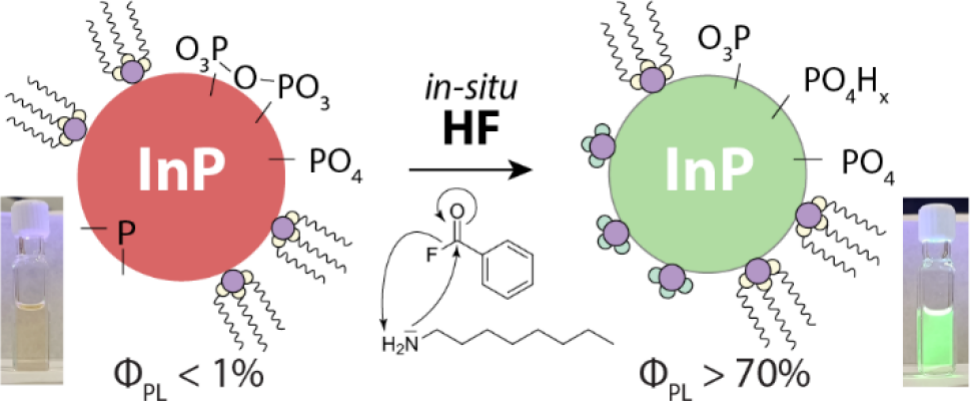

Indium phosphide quantum dots are the main alternative for toxic and restricted Cd-based quantum dots for lighting and display applications, but in the absence of protecting ZnSe and/or ZnS shells, InP quantum dots suffer from low photoluminescence quantum yields. Traditionally, HF treatments have been used to improve the quantum yield of InP to ∼50%, but these treatments are dangerous and not well understood. Here, we develop a postsynthetic treatment that forms HF in situ from benzoyl fluoride, which can be used to strongly increase the quantum yield of InP core-only quantum dots. This treatment is water-free and can be performed safely. Simultaneous addition of the z-type ligand ZnCl_2_ increases the photoluminescence quantum yield up to 85%. Structural analysis via XPS as well as solid state and solution NMR measurements shows that the in situ generated HF leads to a surface passivation by indium fluoride z-type ligands and removes polyphosphates, but not PO_3_ and PO_4_ species from the InP surface. With DFT calculations it is shown that InP QDs can be trap-free even when PO_3_ and PO_4_ species are present on the surface. These results show that both polyphosphate removal and z-type passivation are necessary to obtain high quantum yields in InP core-only quantum dots. They further show that core-only InP QDs can achieve photoluminescence quantum yields rivalling those of InP/ZnSe/ZnS core/shell/shell QDs and the best core-only II–VI QDs.

## Introduction

Colloidal quantum dots (QDs) are promising luminescent materials for use in photonic applications such as LEDs, displays, and lasers.^1−3^ With a tunable band gap in the range of 1.3–3 eV, InP QDs are a ROHS-compliant alternative to traditional Cd-based QDs for visible and near-infrared applications. Core/shell InP structures have become the industry standard for light-emitting applications, but core-only QDs offer advantages such as high absorptivity of blue light and better charge carrier mobilities when used in, for example, electroluminescent devices. However, as-synthesized InP QDs invariably display low photoluminescence quantum yields (PLQYs), usually attributed to surface oxidation,^4−8^ although an atomistic picture of how oxidation results in trap states is missing. Treatments of InP with hydrogen fluoride (HF) have long been used to increase its luminescence,^9−15^ and HF treatment is still employed in the preparation of highly luminescent core/shell InP/ZnSe/ZnS QDs.^6,16,17^ However, the PLQYs of HF treated core InP QDs are typically limited to <50%, and the mechanisms through which HF removes or passivates electronic traps remains unclear. On one hand, the removal of oxidized phosphorus and trap passivation due to surface fluorination have been suggested.^6,16−18^ On the other hand, most studies have employed aqueous HF even though water is a known source of oxidation and can also have detrimental effects on the PLQYs.^19−22^

Here we study the reaction of InP QDs with anhydrous HF produced in situ via the amidation of an acyl fluoride with an alkylamine, which also constitutes a safer alternative to handling HF directly. In this article we refer to this approach as “in situ HF treatment”. The reaction is investigated through a combination of optical and structural techniques including absorption and photoluminescence spectroscopy, X-ray photoelectron spectroscopy (XPS), X-ray diffraction (XRD), and solution as well as solid state nuclear magnetic resonance (NMR). We find that in situ HF quickly etches InP QDs via the formation of PH_3_ and InF_3_, after which InF_3_ binds to the InP surface, passivating dangling phosphorus bonds and increasing quantum yield. Although PLQYs up to 70% can be reached using this in situ HF treatment and 85% when additional ZnCl_2_ z-type ligands are added, solid state NMR results show that oxidized phosphorus species (PO_3_ and PO_4_) remain present on the surface. This suggests that the presence of PO_3_ and PO_4_ on the InP surface does not lead to trap states, which is further supported by DFT calculations. A conversion of polyphosphates to H_*x*_PO_4_ is observed, however, indicating that in situ HF protonates and breaks up polyphosphate species, allowing for further z-type ligand passivation of the surface. InF_3_ and in situ HF treatments of InP QDs with varying degree of oxidation are shown to support this hypothesis.

## Methods

All procedures were executed in an inert atmosphere (Schlenk line or glovebox, H_2_O < 0.1 ppm, O_2_ < 0.1 ppm).

### Materials

The following materials were purchased from Merck Sigma and used as received: indium acetate (99.99%), palmitic acid (PAH, 99%), trioctylphosphine (TOP, 97%), anhydrous acetone (99.8%), octylamine (99%), anhydrous hexadecane (99%), zinc chloride (ZnCl_2_, 99.999%), tributylphosphine (TBP, 97%), and triethylamine (99.5%). Tris(trimethylsilyl)phosphine (TMSP, 98%, Strem; *caution: TMSP is a highly pyrophoric substance that can release toxic phosphine gas upon reaction with air*), anhydrous toluene (99.8%, Alfa Aesar), benzoyl fluoride (98%, TCI), didodecylamine (97%, TCI), and indium fluoride (InF_3_, 99.95%, Alfa Aesar) were used as received. 1-Octadecene (ODE, 90%, Merck Sigma) and mesitylene (98%, Merck Sigma) were degassed in vacuo at 100 °C and room temperature respectively before being stored in a nitrogen-filled glovebox.

### Hot-Injection Synthesis of InP QDs

The synthesis is based on that reported by Won et al.^6^ In a typical synthesis, indium acetate (585 mg, 2.00 mmol), palmitic acid (1535 mg, 6.00 mmol), and ODE (50 mL) were added to a three-neck round-bottom flask. This mixture was degassed in a Schlenk line at 0.1 mbar and 120 °C for 60 min, during which indium palmitate (In(PA)_3_) formed and acetic acid evaporated under vacuum. Nitrogen gas was then blown over the surface through a needle (rate = 0.4 L/min, pressure ca. 1 bar), and the temperature was raised. At 280 °C, a TMSP solution (5 mL of 0.3 M TOP) was injected, causing the temperature to drop instantly. The reaction was allowed to run at 260 °C for 12 min before being cooled by an air gun. At room temperature, a transparent, dark red dispersion was obtained. The quantum dots were purified by precipitation with anhydrous acetone (5 vol equiv) and separated by centrifugation (10 min at 5000 rpm). After carefully discarding the supernatant, the liquid precipitate containing the QDs was diluted in anhydrous toluene (8 mL). The washing procedure was repeated before obtaining the InP QD stock solution. The concentration of this stock solution (ca. 1.37 mM) was assessed via optical absorption considering an extinction coefficient ε of 0.45 cm^–1^ μM^–1^ at 330 nm, which was determined via the Maxwell–Garnett model developed for QDs.^23^

### Heat-Up Synthesis of InP QDs

This procedure is based on the work of Li et al.^24^ Indium acetate (436 mg, 1.5 mmol), palmitic acid (999.9 mg, 3.9 mmol), and ODE (41.4 g, 52.5 mL) were combined in a three-neck round-bottom flask. The mixture was connected to a Schlenk line, and N_2_ was bubbled through the solution at a rate of 0.3 L/min. The mixture was heated at 150 °C for 30 min. During this time, indium palmitate (In(PA)_3_) was formed, and acetic acid was evaporated away by the gas stream. Then, (i) TOP (6.23 g, 7.5 mL) and (ii) TMSP (187 mg, 0.75 mmol) in ODE (9.47 g, 12 mL) were sequentially injected into the In(PA)_3_ solution, and the temperature was increased to 270 °C and kept at that value for 5 min. The flask was then cooled by air gun to 200 °C, after which a water bath was used to quickly bring it to room temperature, at which point a dark red InP quantum dot dispersion was obtained. The quantum dots were purified in the same way as stated above for the hot injection method.

### In Situ HF Treatment

*Caution: because toxic phosphine gas is formed during this treatment, it should only be performed inside a glovebox or well-ventilated fume hood.* In a typical treatment, the InP QD stock dispersion (30 μL, 41 nmol) was first diluted in mesitylene (820 μL) inside a glass vial. The mixture was heated to 150 °C, at which point an (i) octylamine solution (90 μL of 0.64 M mesitylene, 58 μmol) and a (ii) benzoyl fluoride solution (90 μL of 0.45 M mesitylene, 41 μmol) were sequentially added. The addition of the benzoyl fluoride triggers a color change in the QD solution from orange to yellow, and formation of a colorless vapor can be observed. After 2 min, the vial caps were closed, and the mixture was allowed to react at 150 °C for another 58 min. For the treatment at 200 °C, mesitylene was replaced by hexadecane and octylamine was replaced by didodecylamine. Additionally, the injected volumes of benzoyl fluoride and amine solutions were increased to 130 μL, and the cap was added immediately after injection (of benzoyl fluoride) to minimize the effect of evaporation. The quantum dot mixture was then purified through a filter (0.2 μm PFTE), after which 10–20 vol equiv of anhydrous acetone was added to precipitate the QDs. The mixture was centrifuged for 10 min at 6000 rpm, after which the yellow-green precipitate was redispersed in toluene.

### ZnCl_2_ Addition

A ZnCl_2_ solution (10 μL of 0.64 M mesitylene, 6.4 μmol) was added to the QD dispersion before starting the in situ HF treatment which was otherwise performed as stated above, except the added volumes of benzoyl fluoride and amine solution were increased to 150 μL and the treatment only lasted 5 min. The ZnCl_2_ solution (0.64 M mesitylene) was prepared by mixing ZnCl_2_ (436 mg, 3.2 mmol), TBP (2266 mg, 11.2 mmol), and mesitylene (to a volume of 5 mL) under stirring until fully dissolved. TBP was necessary to dissolve the ZnCl_2_ in polar solvents and accurately determine the concentration.

### Pure InF_3_ Treatment

InF_3_ (50 mg, 0.29 mmol), mesitylene (1 mL), and the InP QD stock dispersion (30 μL, 41 nmol) were combined in a glass vial. The mixture was stirred at 150 °C for 1 h.

### Optical Characterization

UV–vis spectra were recorded on a PerkinElmer Lambda 365 spectrometer. Fluorescence measurements were recorded on an Edinburgh Instruments FLS980 spectrometer equipped with a PMT 400 detector. Photoluminescence quantum yields were measured in accordance with IUPAC methodology^25^ against a coumarin 102 dye solution in ethanol (purity >99.8%) at an excitation wavelength of 387 nm (OD at 387 nm for all samples ∼0.1). Integrated emission intensities were corrected using a detector calibration curve. Measuring the coumarin 102 quantum yield in an integrating sphere in the same setup gave a value of 99%, but to calculate the quantum yield, the literature value of 95% was considered for the quantum yield of coumarin 102.^26^ Additionally, a typical in situ HF + ZnCl_2_ treated InP sample was measured in an integrating sphere in the same instrument to have a PLQY of 84% (Figure S19), confirming the values obtained in the dye measurements. PL decay traces were collected on a Edinburgh Instruments Lifespec TCSPC setup with a 400 nm pulsed laser. The emission was measured at 540 nm. TRPL traces were fitted with a biexponential fitting curve, after which intensity-weighted average lifetimes were calculated by the following equation: τ_ave_ = (*A*_1_τ_1_^2^ + *A*_2_τ_2_^2^)/(*A*_1_τ_1_ + *A*_2_τ_2_), where *A*_*n*_ and τ_*n*_ are the *n*th amplitude and lifetime parameters obtained from the biexponential fit.^27^

### X-ray Diffraction (XRD)

Samples were prepared by dropcasting QD dispersions on zero-diffraction silicon substrates. Diffraction patterns were recorded using a Bruker D8 Advance diffractometer (Cu Kα, λ = 0.15406 nm).

### X-ray Photoelectron Spectroscopy (XPS)

Samples were prepared by dropcasting the QD dispersions onto thin aluminum substrates inside a nitrogen-filled glovebox and were vacuum-transferred to the instrument to avoid exposure to air. Measurements were performed under UHV (<2 × 10^–7^ mbar) on a ThermoFisher K-Alpha equipped with an Al Kα source, radiating with an energy of 1486 eV. A flood gun (Ar) was active during all measurements to prevent charging of the samples.

### Solution Nuclear Magnetic Resonance (NMR)

Solution NMR spectra were recorded on an Agilent 400-MR DD2 equipped with a 5 mm ONE NMR Probe and operating at 25 °C. ^1^H NMR (399.7 MHz) spectra were collected with a recycle delay of 1 s in deuterated toluene. Signals were referenced according to the residual methyl peak of toluene-*d*_8_ (2.08 ppm). ^31^P NMR spectra (161.8 MHz) were collected a recycle delay of 1 s in toluene (enriched with toluene-*d*_8_). ^31^P signals were externally referenced to H_3_PO_4_.

### Solid State Nuclear Magnetic Resonance (ssNMR)

Samples were loaded into 4 mm zirconia rotors inside a nitrogen-filled glovebox (O_2_ < 0.1 ppm, H_2_O < 0.1 ppm). Measurements were performed using an Agilent 400 MHz magnet operating at ^1^H, ^31^P, and ^13^C resonance frequencies of 399.9, 161.9, and 100.6 MHz, respectively, using a CMX 4.0 mm Triple Res T3 SPC400-550. The MAS frequency was set to 12.5 kHz for all measurements. One-pulse ^1^H MAS spectra were collected with a recycle delay (*d*_1_) of 30 s and a 2.5 μs pulse width. One-pulse ^31^P MAS spectra were collected with a recycle delay (*d*_1_) of 10–15 s and a 3.4 μs pulse width. ^1^H → ^31^P CPMAS measurements were performed with a ^1^H π/2 pulse length of 3.4 μs, a CP period of 2 ms, and a recycle delay (*d*_1_) of 3 s. ^1^H → ^13^C CPMAS measurements were performed with a ^1^H π/2 pulse length of 3.4 μs, a CP period of 1.2 ms, and a recycle delay (*d*_1_) of 3 s. During all CPMAS measurements, proton decoupling was performed using the Spinal-64 decoupling sequence. ^31^P{^1^H} heteronuclear correlation (HETCOR) measurements were performed with a CP contact time of 2 ms. For each of the 512 transients in the ^1^H dimension, 2048 ^31^P scans were accumulated. A recycle delay of 3 s was applied after each scan. Spectra were referenced to external H_3_PO_4_.

Additional ssNMR spectra were collected with a Bruker Ascend 500 magnet (11.7 T) equipped with a NEO console operating at ^1^H and ^31^P resonance frequencies of 500.16 and 202.45 MHz, respectively, using a 4 mm three-channel DVT MAS probe head from Bruker. Samples were filled into 4 mm zirconia rotors in an argon-filled glovebox (O_2_ < 0.1 ppm, H_2_O < 0.1 ppm). InP samples were impregnated into an Al_2_O_3_ filler. The MAS frequency was set to 8 kHz for all measurements. One-pulse ^31^P MAS spectra were collected with a recycle delay of 50 s and a 5 μs pulse width. ^1^H → ^31^P CPMAS measurements were performed with a ^1^H π/2 pulse length of 3.85 μs and a CP period of 500 μs. A total of 30000 scans were accumulated with a recycle delay of 2 s. Proton decoupling was performed during acquisition using the Spinal-64 decoupling sequence.

## Results and Discussion

### In Situ HF Treatment

**Scheme 1:**
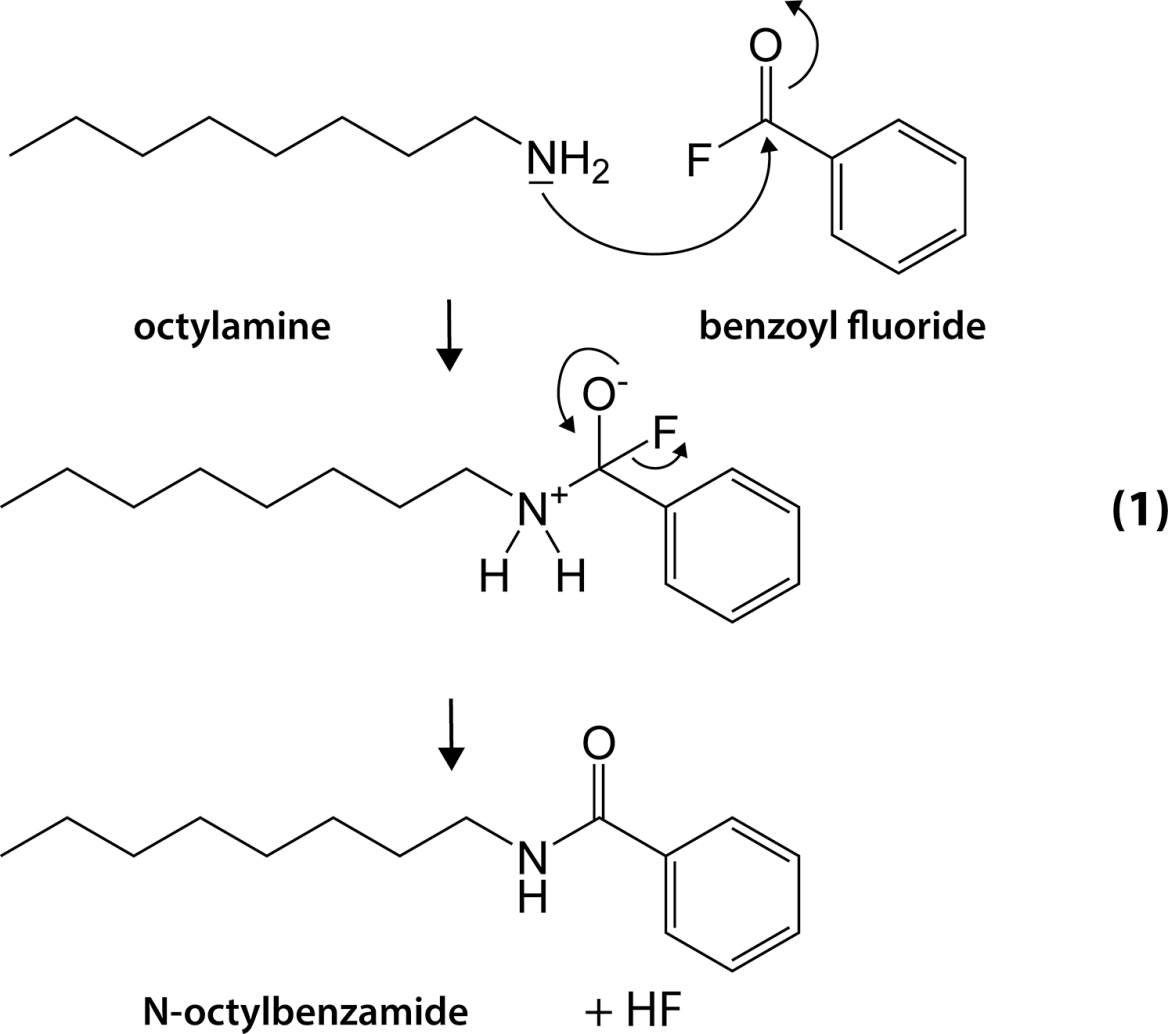
Amidation of Octylamine and Benzoyl Fluoride Leads to the Formation of *N*-Octylbenzamide and Hydrogen Fluoride (HF)

Because standard HF treatments pose a considerable safety hazard and expose the InP QDs to water, we searched for a safer method to deliver HF in an anhydrous way, which could be performed entirely within a glovebox. Herein, we exploit the amidation of acyl fluorides to generate HF in situ, which is considered to be a rapid exothermic reaction.^28^ In particular, benzoyl fluoride is reacted with octylamine in mesitylene. Benzoyl fluoride’s phenyl group delocalizes electron density, which makes it highly electrophilic. When combined with octylamine, amidation takes place upon which *N*-octylbenzamide and hydrogen fluoride are formed via reaction 1 depicted in Scheme 1. Although this treatment is performed postsynthesis, we will refer to it as “in situ HF treatment” to differentiate from regular HF treatments because the HF is not added directly to the QD solution but is formed only after the amidation reaction.

**Scheme 2:**
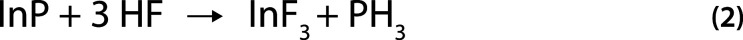
Proposed HF Etching Reaction of InP

The formation of *N*-octylbenzamide after reaction 1 is confirmed using ^1^H NMR as shown in Figure S1 of the Supporting Information. When the reaction occurs in the presence of InP QDs (diameter = 2.6 nm), PH_3_ is formed as confirmed by solution ^31^P NMR (Figure S2) and InF_3_ is observed as a white precipitate (confirmed by XPS in Figure S3). We propose that the InP nanoparticles are etched by the HF according to the simple reaction 2 in Scheme 2, wherein InF_3_ and PH_3_ are formed.

**Figure 1:**
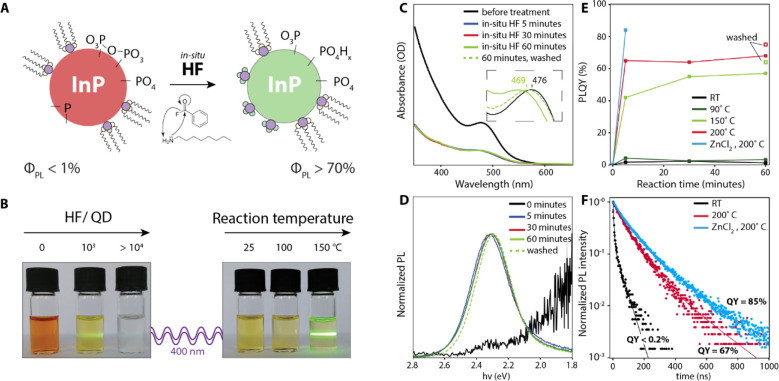
Schematic representation and optical characteristics of oxidized InP QDs treated with in situ HF. As illustrated in panel A, InP QDs are reacted with HF produced in situ via the amidation reaction of benzoyl fluoride and octylamine in anhydrous conditions. (B) Photographs of InP QD samples treated with in situ HF at various concentrations and temperatures reveal that in certain conditions highly luminescent samples can be obtained; however, strong material losses are also apparent. An optical characterization of aliquots collected over the course of a typical in situ HF treatment at 150 °C is provided in panels C–E, namely, (C) absolute absorption spectra (inset shows normalized absorption spectra before and after the treatment), photoluminescence (D) spectra (λ_ex_ = 387 nm), and (E) PLQYs. In panel E, we also report the evolution of PLQYs at other reaction temperatures. In the untreated QDs, a weak band edge luminescence and some trap emission can be observed. Upon HF treatment a strong drop in absorption quickly occurs, and only at reaction temperatures of 150 °C or higher is a strong increase in quantum yield observed. The addition of ZnCl2:TBP complexes during the treatment leads to even higher quantum yields. Panel F shows the time-resolved PL lifetime measurements of samples before and after the in situ HF treatment. Solid lines show the biexponential fits.

At intermediate concentrations (around 1000 molecules of benzoyl fluoride per QD), the sample is only partially etched, and highly luminescent InP QDs can be obtained (Figure 1B). Figure 1C shows the absorption spectra of the InP QDs over the course of a typical in situ HF treatment. Strong etching immediately takes place upon injection of benzoyl fluoride into the octylamine solution containing the QDs, leading to a visible decrease in optical density (Figure 1C). The drop in optical density is observed to increase with increasing HF concentration, as can be seen in Figure S4, and is in line with a net loss of InP material in accordance with reaction 2 in Scheme 2. Furthermore, the first absorption peak is initially found slightly blue-shifted (from 476 to 463 nm) and then red-shifts over time as the treatment is continued, suggesting that the particles regrow after the initial etching.

Figures 1D and 1E show the effect of the in situ HF treatment on the photoluminescence (PL) of the InP QDs. Running the treatment at 150 °C for up to 1 h increases the PL quantum yield (PLQY) to 55% and reduces the relative intensity of red tail in the PL, attributed to trap emission.^29−31^ Full width at half-maxima after the in situ HF treatment were ∼70 nm (300 meV), which is typical for InP after HF treatment^9,18,32^ and wider than the narrowest emission observed for core InP.^17^ Purification of the treated QDs leads to size-selective precipitation (as can be seen by the red-shift in absorption in the inset of Figure 1C) and to an increase in the PLQY to 64%. Although the quick drop in absorption that happens at the start of the treatment appears temperature-independent, the effect of the HF treatment on the PLQYs appears to be strongly temperature related. As can be seen in Figure 1E, both at room temperature and at 90 °C, PLQYs never exceeded 5% regardless of treatment time. At 150 °C a quick rise in PLQY to ca. 40% is observed over the first few minutes, and thereafter the PLQY keeps on increasing over time. At 200 °C even higher quantum yields (>70%) are observed, and PLQY evolution appears complete in under 5 min. This shows that there is a sizable activation energy for restructuring the surface, possibly related to the removal of the original ligands. We note that the in situ HF treatment reproducibly results in PLQY values of ∼70% but that a further increases is possible by adding additional ZnCl_2_ z-type ligands to the treatment, which increases the PLQY further to 85%. The addition of ZnCl_2_ will be discussed in more detail below. Time-resolved PL measurements (Figure 1F) show that longer PL lifetimes are obtained for samples with higher quantum yield. Average lifetimes (see the Methods section for details) increased from 29 ns in untreated QDs to 107 ns after the in situ HF treatment and 138 ns when ZnCl_2_ was added during the treatment.

To disentangle the various chemical processes involved in the in situ HF method, we conducted a series of control experiments. First, the effects of treating InP QDs with either only octylamine or benzoyl fluoride were investigated and are summarized in Figures S5 and S6. Octylamine barely affects the QDs on its own (Figure S5). Benzoyl fluoride treatments, however, were often found to cause the QDs to precipitate from the mesitylene dispersion, which we assign to a reaction of benzoyl fluoride with palmitate ligands, forming anhydrides and fluoride ions (Figure S6). This reaction appears to be rather slow with respect to amidation and, unlike the in situ HF reaction, does not improve the luminescence.

Furthermore, treatments similar to the in situ HF treatment with benzoyl bromide (forming in situ HBr) and benzoyl chloride (forming in situ HCl) are reported in Figure S7. Neither HCl nor HBr increases the PLQY of InP QDs, and at higher concentration they quickly cause full dissolution of all nanocrystals. Treatments with organic Brønsted acids were also attempted (Figure S8) but only resulted in small increases in PLQY of 1–2%. It thus seems that the etching reaction 2 can be extended to a variety of protic acids, but among these, only HF has the ability to increase the PLQY of InP quantum dots.

We consider that InF_3_, which forms during the HF treatment according to reaction 2, could play an active role in the trap passivation process as a z-type ligand. Interestingly, treating InP QDs with solid InF_3_ powder (hereafter termed “pure InF_3_ treatment”) increases the PLQY up to 28% (Figure S9). This observation was also reported by Kim et al. as well by Calvin and colleagues, although they attempted to dissolve InF_3_ in TOP and tetrahydrofuran, respectively.^18,33^ We note that after our pure InF_3_ treatment, the absorption drop that is typical for the in situ HF treatment is absent. However, the QDs precipitate out of dispersion at high concentrations of InF_3_. Furthermore, this treatment also appears temperature activated; only at 150 °C is an increase in quantum yield observed.

Taken together, these observations suggest that several trap passivation mechanisms might be at play during the HF treatment, including passivation or removal of dangling bonds, as well as the potential changes in the surface oxide species. To elucidate structural changes occurring during this in situ HF treatment, we performed a series of analyses.

### Structural Analysis

To obtain further information about the processes responsible for the increase in photoluminescence quantum yield, X-ray diffraction (XRD), X-ray photoelectron spectroscopy (XPS), and solution as well as solid state nuclear magnetic resonance (NMR) analyses were performed. The XRD patterns of the QDs before and after treatment are shown in Figure S10. In both diffractograms peaks that can be indexed to zinc blende InP are observed as well as a peak at 20° arising from the palmitate ligands.^34^ The latter peak is strongly reduced after the in situ HF treatment, suggesting that the in situ HF treatment leaves the InP crystal lattice intact while inducing the removal of a large fraction of the palmitate ligands, in agreement with previous observations by Calvin et al. after their HF treatment.^34^

**Figure 2:**
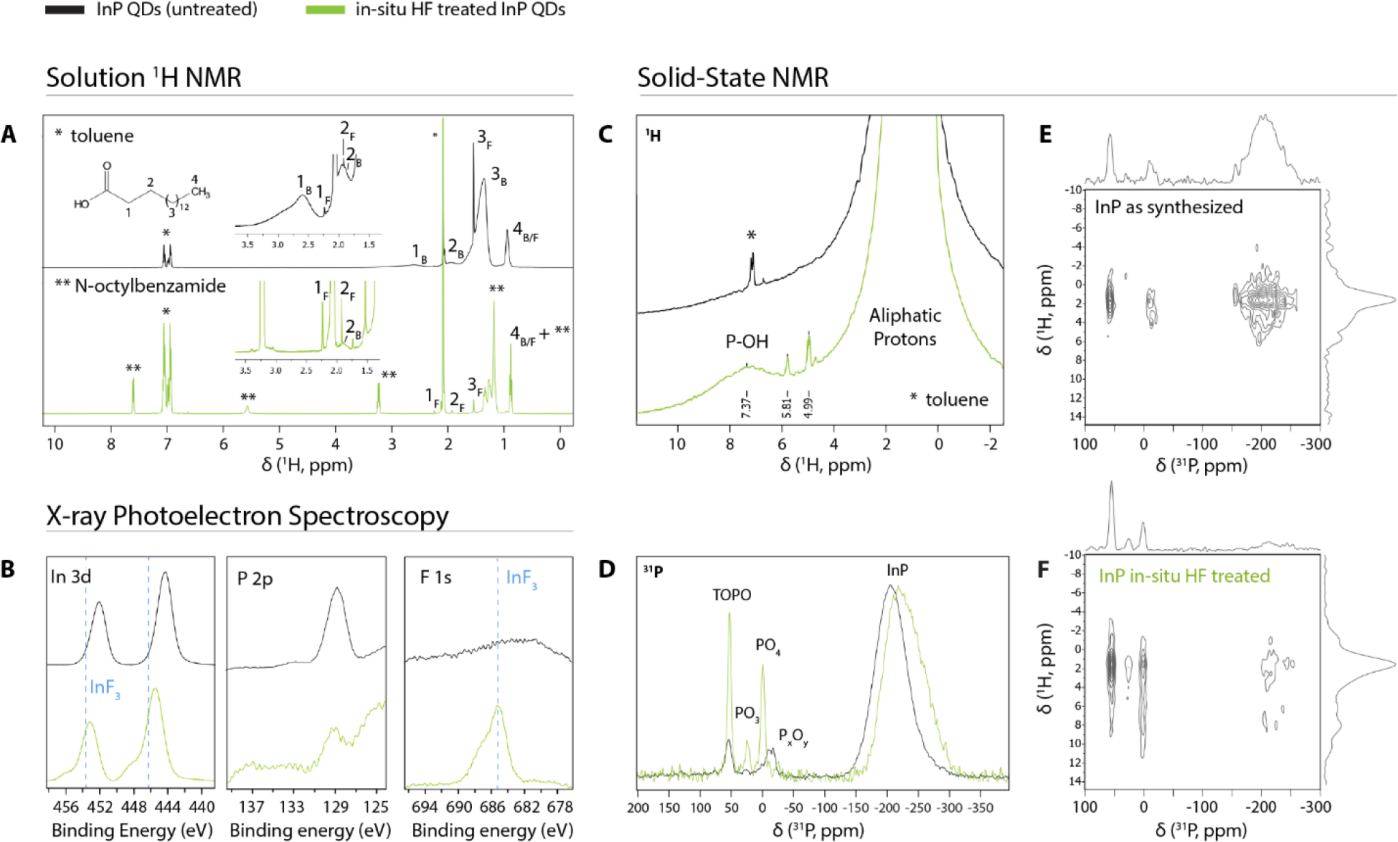
Structural characterization of oxidized InP QDs treated with in situ HF. (A) Solution ^1^H nuclear magnetic resonance (NMR) spectra of InP QD dispersions before and after treatment with in situ HF (solvent: deuterated toluene). Peaks corresponding to palmitate ligands are labeled “B” for species bound to the nanocrystal surface and “F” for free species. A reduction of the bound palmitate fraction is observed after the treatment. (B) XPS spectra of InP QDs before and after the in situ HF treatment. Part of a plasmon loss peak of the aluminum substrate is visible in the P 2p part of the spectrum at lower binding energies. Some of the InP has been converted to InF_3_ during the treatment. InF_3_ reference peaks were copied from XPS data by Kim et al.^18^ (C–F) Solid state NMR spectra of InP QDs before and after the in situ HF treatment. (C) ^1^H single pulse. (D) ^31^P single pulse. Spectra are normalized to the intensity of the core InP peak to highlight relative differences between the two samples. (E) ^31^P{^1^H}cross-polarization HETCOR of the QDs before treatment. (F) ^31^P{^1^H} cross-polarization HETCOR of the QDs after the in situ HF treatment. A CP time of 2 ms was used for both HETCORs. Average ^1^H and ^31^P spectra are plotted at the top and left sides. The MAS frequency was 12.5 kHz for all measurements.

The removal of surface ligands is further confirmed using solution ^1^H NMR (Figure 2A). In solution NMR, ligands that are bound to the NC surface exhibit broad peaks due to their slow rotational motion (slow tumbling).^35,36^ As shown in Figure 2A, such peaks are clearly seen at 2.60, 1.93, 1.35, and 0.93 ppm for the untreated sample and belong to the α, β, remaining chain, and methyl tail protons of the palmitate ligands, respectively. In addition, some free palmitate is observed as narrow lines at similar resonances. After the HF treatment the same peaks are visible, but the intensity of free palmitate relative to bound palmitate has strongly increased, indicating that palmitate ligands have been removed from the InP QD surface.

XPS spectra of the samples are shown in Figure 2B. In the spectra before the treatment, typical InP peaks can be observed at 444.4 and 452.2 eV (In 3d) and at 128.8 eV (P 2p). After the treatment, the indium peaks have shifted to higher binding energies, and a peak is observed at 485.2 eV corresponding to F 1s in InF_3_. This shows that part of the InP has been converted to InF_3_, in accordance with reaction 2. This InF_3_ then likely binds to the InP surface during the treatment, replacing In(PA)_3_ units. Elemental quantification of the XPS data shows that the P:In ratio is maintained at 1:2, which is typical for InP QDs of this size and shows that both the starting and the treated QDs have an indium-rich surface.^37^ A F:In ratio of 2:1 is also found. The unchanged P:In ratio indicates that fluoride does not replace phosphide but rather is bound to indium atoms at the surface of the QDs.

**Scheme 3:**
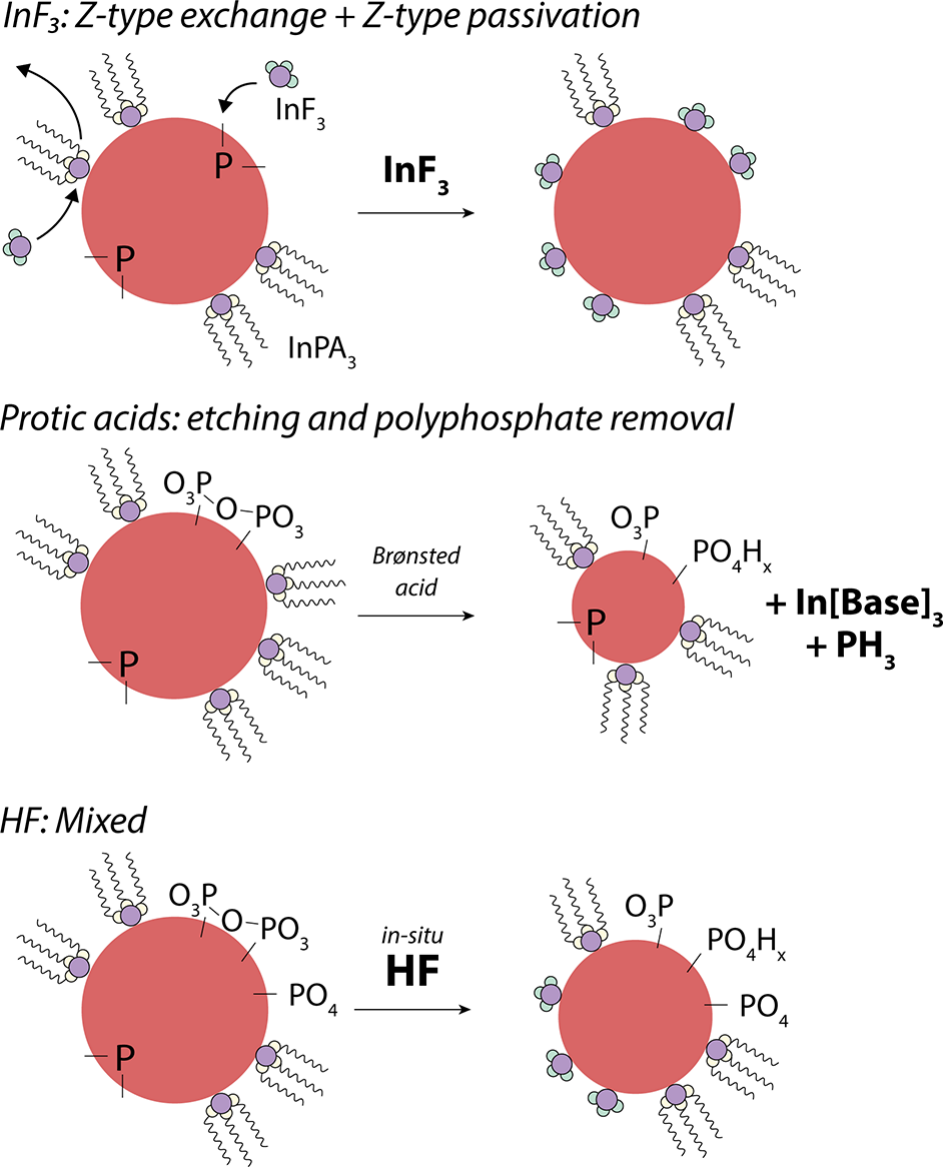
Reaction Schemes of Three Different Type of Surface Treatments of InP QDs

Taken together, these results show that after the treatment palmitate ligands have been removed from the surface and have been replaced with fluoride. Kim and colleagues suggested that the replacement of palmitate with fluoride ions upon HF treatment takes place on the surface of the QDs as the result of a direct acid–base reaction between HF and palmitate.^18^ We do not exclude that part of the exchange may take place during the initial etching according to this mechanism, where HF can directly protonate palmitate and form InF_3_ on the surface. However, after the initial etching has finished and all the HF has reacted, the additional increase in PLQY should be attributed to the partial exchange of In(PA)_3_ with the InF_3_ that was formed in a z-type exchange mechanism, as depicted in Scheme 3. This hypothesis is in line with the slow and thermally activated increase in PLQY (Figure 1D). The results from the pure InF_3_ treatment (Figure S9) show that while the solubility of InF_3_ in mesitylene is presumably low, the exchange of In(PA)_3_ with InF_3_ can still be facilitated at 150 °C. However, on these QDs, the increase in PLQY after the pure InF_3_ treatment is always significantly lower than for in situ HF treatment, pointing to additional benefits of HF on the InP surface, related to the removal of surface oxides, as discussed next.

### Solid State NMR Characterization

Solid state NMR (ssNMR) is one of the most powerful tools to investigate the atomic structure of InP QDs and has been used often to investigate oxidized phosphorus species.^22,37−40^ Through magic angle spinning (MAS), phosphorus and hydrogen species that are in the solid phase and therefore severely broadened in solution NMR can be observed and distinguished. Single-pulse spectra give quantitative information about the species that are present, while surface species can be identified by utilizing a cross-polarization (CP) pulse sequence to transfer magnetization from the hydrogen atoms in the ligands to the phosphorus species on the surface. 2D ssNMR techniques such as heteronuclear correlation (HETCOR) can also be performed to obtain information about the spatial proximity of specific species.

Figures 2C–F show various ssNMR measurements on InP QDs before and after the in situ HF treatment. The ^1^H solid state spectra are expected to resemble those observed in solution NMR. However, significant broadening should be expected in the solid state spectra because molecular motion is greatly reduced in the solid state, which cannot be fully compensated for by magic angle spinning. This is indeed observed in the ^1^H solid state spectrum of the untreated QDs (Figure 2C): apart from a small residual solvent peak, only aliphatic resonances are observed, ascribed to palmitate ligands, similar to the solution ^1^H spectrum (Figure 2A). After the HF treatment, additional weaker resonances arise: two sharp ones at 5.0 and 5.8 ppm (ascribed to residual trace water and *N*-octylbenzamide, respectively) and another centered at 7.4 ppm. The latter is much broader than the other two, indicating strong bonding to the surface. Baquero and colleagues earlier observed a peak at a similar frequency, which they ascribed to P–OH with the help of forward and back cross-polarization (FBCP) experiments.^40^ This species is invisible in solution NMR due to its strong bonding to the surface and low intensity.

In Figure 2D, the ^31^P ssNMR spectra before and after treatment are presented. The core phosphorus atoms in InP QDs are known to give a broad signal around −200 ppm.^22,37−40^ This broad signal is indeed observed in the QDs before the treatment and shifts from −198 to −223 ppm upon treatment with HF. We ascribe this shift to the exchange of InPA_3_ for InF_3_. DFT studies on small InP complexes provide theoretical support for this observed frequency shift (Figure S11). Further downfield in Figure 2D resonances can be observed between −20 and +60 ppm. These peaks are enhanced when cross-polarization from hydrogen is employed (Figure S12), thus confirming that they are present on the surface, close to the hydrogen atoms of the palmitate ligands. These resonances have often been observed in ^31^P ssNMR spectra on InP QDs and are usually attributed to various surface–oxide complexes. Specifically, the peaks around 0 and 24 ppm are assigned to P(V) (i.e., PO_4_^3–^) and P(III) (i.e., PO_3_^–3^) complexes^22,37,38^ or trioctylphosphine oxide (TOPO),^41^ while the peak at 53 pm is assigned either to PO_2_^3– ^^22^ or to TOPO.^38,39^ Because trioctylphosphine (TOP), which can readily be oxidized to TOPO, is employed in our synthesis, we cannot unambiguously assign these observed surface peaks based on only the single pulse and cross-polarization spectra. 2D ^31^P{^1^H} HETCOR experiments were therefore performed to help identity the nature of the surface phosphorus resonances. Using this method, phosphorus and hydrogen resonances that have the ability to cross-polarize with each other are selectively shown. In other words, only phosphorus species that are spatially close to hydrogen and vice versa appear in the spectra. A correlation peak between two specific species in the 2D spectrum indicates that those species are spatially close to one another.

^31^P{^1^H} HETCOR on the as-synthesized particles (Figure 2E) reveals correlations between all phosphorus peaks and the aliphatic hydrogens. No strong correlations can be distinguished between any specific species. This shows that all surface phosphorus species have approximately the same spatial proximity to hydrogen atoms on the ligands.

After the in situ HF treatment, however, two specific correlations can be distinguished (Figure 2F). The aliphatic protons correlate with the phosphorus peak at 53 ppm, and the new hydrogen peak at 7.4 ppm which appears after the treatment correlates with the phosphorus peak at 0 ppm. The correlations become even more clear when looking at single slices of the 2D spectrum, as shown in Figure S13. These two correlations show spatial proximity of the aforementioned proton and phosphorus species and can help identify the nature of the phosphorus peaks.

First, the peak around 0 ppm is usually ascribed to PO_4_ species.^22,37,42^ In the untreated QDs, this peak is positioned more upfield, at −15 ppm. This resonance is typically associated with pyro- or polyphosphates.^43,44^ Polyphosphates are expected to form on InP surfaces upon oxidation at temperatures around 573 K^19^ and could thus be present after InP hot injection synthesis. Upon treatment the resonance narrows down and shifts to 0 ppm, nearly the same value as the chemical shift of the H_3_PO_4_ reference. Our results thus suggest that polyphosphate species are formed during the high-temperature synthesis, after which they are broken up and protonated during the HF treatment, resulting in H_*x*_PO_4_ species and the correlating P–OH peak in the hydrogen spectrum at 7.4 ppm. Second, the peak at 24 ppm is usually assigned to PO_3_ species.^22,37,38^ DFT calculations are in line with this assignment of PO_4_ and PO_3_ and support the general trend that PO_*x*_ species appear at more positive chemical shifts for lower *x* (Figure S11).

Although these DFT calculations suggest that the final peak at 53 ppm may be ascribed to PO_2_, we argue that it should instead be assigned to TOPO that is formed during the synthesis and bound to the surface of InP QDs, in line with the assignment made by Stein and colleagues.^38^ The resonance at 53 ppm is almost identical with that of free TOPO (46 ppm, Figure S12). This assignment of TOPO is further supported by additional ssNMR spectra that were obtained after InP syntheses with and without the use of TOP (Figure S12). If no TOP is used in the synthesis, no peak is observed at 53 ppm.

After the in situ HF treatment we observe an increase in the intensity of the resonances in the 60 to −20 ppm range compared to the broad core phosphide peak around −200 ppm (Figure 2D). By normalizing both spectra to their respective InP core peak area, the integrals of each species relative to the core InP peak were calculated. Relative integrals of the PO_4_ and PO_3_ peaks increase from 0.05 to 0.07 and from 0.01 to 0.02, respectively, after the in situ HF treatment. This shows that, surprisingly, the in situ HF treatment does not result in a net decrease of the ratio of surface oxidized phosphorus/unoxidized core InP species.

In summary, from ssNMR measurements we observe TOPO, PO_3_ and polyphosphate species on the as-synthesized InP surface. While the in situ HF treatment does not remove all PO_*x*_ species, it breaks up polyphosphates into smaller H_*x*_PO_4_ species.

### Reasons for PLQY Increase

On the basis of the aforementioned XPS and NMR results, the first clear effect of the in situ HF treatment is fluorination of the InP surface. Fluorination of InP QDs has been reported many times, and it is widely accepted that fluorination of InP QDs leads to an increase in the luminescence efficiency.^9,12,18,45,46^ However, multiple different explanations have been given for this relationship. The first mechanism that was proposed to be responsible for the PLQY increase after the HF treatment was reported by Adam et al.,^12^ who suggested that the passivation of phosphorus dangling bonds by fluoride was the main drive of the increase in PLQY. We support a similar mechanism but propose that InF_3_ units bind the dangling phosphorus bonds as z-type ligands rather than fluorine alone. Indeed, DFT calculations show 2-coordinated phosphorus atoms on InP present in-gap states and also that z-type passivation removes these in-gap states.^47^ The efficiency of InF_3_ compared to the other z-type ligands such as In(PA)_3_ or InCl_3_ may be explained by considering steric effects:^48^ bulky species may limit surface coverage of InP QDs. Exchanging part of the indium palmitate for less bulky species like InF_3_ allows a higher surface coverage in z-type ligands and thus a decrease in the number of phosphorus dangling bonds. We note that other works have also proposed that the strong electronegativity of surface fluoride ions could also be related to the high quantum yields.^18,49^

Despite the presence of PO_4_ and PO_3_ surface species after the in situ HF treatment we observe quantum yields >50%. This seems to contradict reports claiming that removal of oxygen is the most important function of the HF treatment that leads to better luminescence properties.^6,16,17^ However, we do observe the removal or conversion of polyphosphates after the in situ HF treatment. This suggests that only certain types of oxide species, i.e., polyphosphates, hamper the quantum yield. The presence of polyphosphates could introduce trap states directly (i.e., orbitals of the polyphosphate species act as traps) or could inhibit full passivation of dangling P with z-type ligands due to their bulky nature. In both cases, polyphosphate removal may be the second important effect of the in situ HF treatment that leads to higher PLQYs. Compared to traditional HF treatments,^9,11,12,18^ our in situ HF treatment appears to yield QDs with higher PLQYs. This could be due to the anhydrous nature of the treatment, which limits oxidation of the QDs due to water and thus the formation of new polyphosphates.

**Figure 3:**
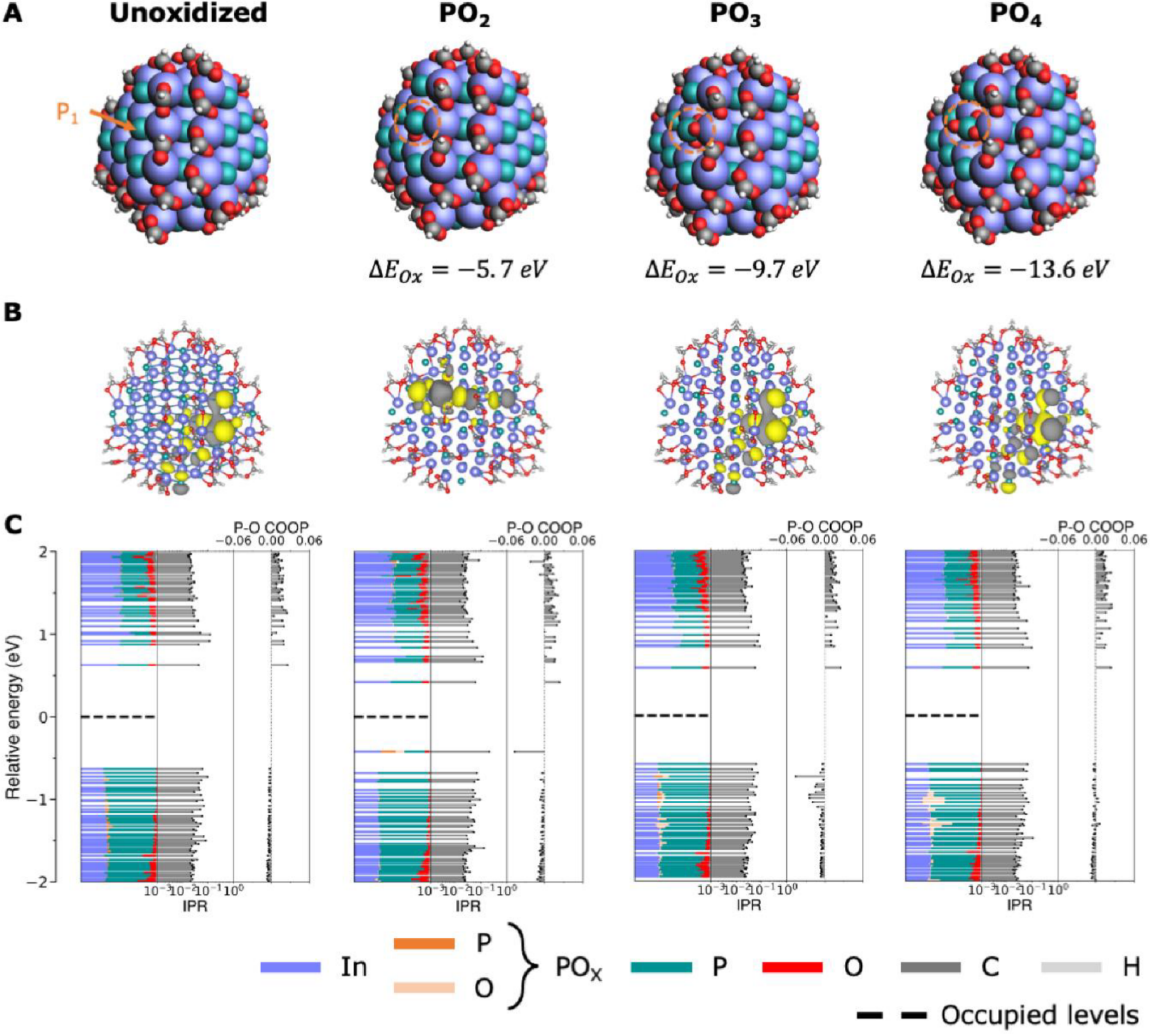
Electronic structure (DFT) calculations on InP QDs with surface PO_*x*_^3–^ species (*x* = 2, 3, 4). (A) Structural composition of each system. (B) Contour plot of the highest occupied molecular orbital (HOMO) of each system. (C) Calculated density of states (DOS), inverse participation ratio (IPR), and crystal orbital overlap population (COOP) of each system.

To test if various PO_*x*_ species on the surface cause in gap states, we performed DFT calculations on QD model systems, replacing surface P^3–^ species with PO_*x*_^3–^, with *x* = 2, 3, and 4 (Figure 3). We find that InP QDs with PO_3_^3–^ or PO_4_^3–^ on the surface do not present states in their band gap, even if a large amount (6 units) of PO_4_^3–^ is added. This is in line with the observation that high quantum yields can be obtained after the in situ HF treatments even though PO_3_ or PO_4_ species remain present on the surface and with observations of high quantum yields in InP core/shell structures despite the presence of interfacial PO_4_.^42,50^ In gap states are observed for PO_2_^3–^ species and assigned to the HOMO orbital of the PO_2_^3–^ complex, formed by an antibonding combination of the P 3p and O 2p atomic orbitals (Figure S14). However, as discussed in the section above, there is no real evidence from the ssNMR spectra that PO_2_^3–^ species are present. Rather, the peak at 53 ppm is assigned to TOPO at the surface. It thus seems more likely that the polyphosphates observed at −15 ppm in the ssNMR spectra of the untreated QDs are associated with trap formation and lower quantum yields. DFT calculations were attempted using P_2_O_7_ as a model polyphosphate species but proved inconclusive in showing a correlation between these species and in-gap states (Figure S15). Negatively charged or neutral P_2_O_7_ species led to n-doping of the quantum dots and reduction of surface indium, similar to earlier results on CdX QDs.^51,52^ This resulted in many in-gap states but could be an artifact of the simulation. Additionally, P_2_O_7_ species appear unstable on the InP surface in simulations, leading to surface reconstructions. A clear atomistic picture of the exact species and arrangement of polyphosphates on the InP QD surface would thus be required to obtain more conclusive information from DFT simulations.

**Figure 4:**
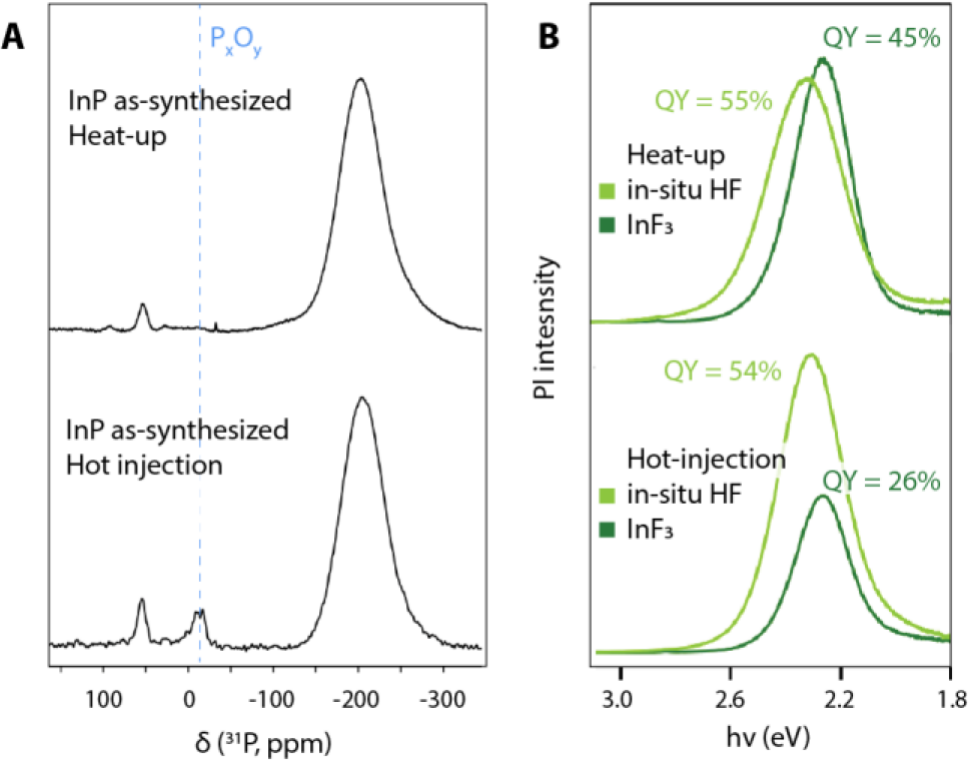
Effectiveness of the in situ HF and of the InF_3_ treatments on InP QDs with varying degree of oxidation. (A) ^31^P ssNMR spectra and (B) photoluminescence spectra and quantum yields. QDs synthesized after the heat-up synthesis do not exhibit a polyphosphate peak around −15 ppm in their ^31^P ssNMR spectra. For these quantum dots, the InF_3_ treatment is almost as effective as the in situ HF treatment in terms of (B) photoluminescence, while a clear difference between the two treatments can be observed for the polyphosphate-containing sample synthesized via hot injection.

Nevertheless, if indeed the polyphosphate species with their peak at −15 ppm are responsible for the low PLQY of the as-synthesized InP QDs, it may be expected that higher efficiencies could be reached by performing the pure InF_3_ z-type treatment on QDs without any of these species present on the surface. It is known that the use of carboxylate precursors can lead to water formation during high temperature InP synthesis, resulting in surface oxidation.^37^ In an attempt to avoid water formation, we synthesized InP QDs via a heat-up method, which resulted in nearly oxide-free particles. These were then treated with anhydrous InF_3_. The results of this experiment are summarized in Figure 4 and are compared with those obtained with the more oxidized particles discussed so far. In Figure 4A, the ^31^P ssNMR spectra of both particles are presented. It is clear that the polyphosphate peak at −15 ppm is only present in the sample synthesized via hot injection and absent in the heat-up sample. When applying the pure InF_3_ treatment to both particles, we observe significantly higher quantum yields for the heat-up sample, up to 45%, vs <28% for the hot injection sample (Figure 4B). These results together with the ssNMR spectra in Figure 2 indicate that the removal of polyphosphates in addition to z-type ligand passivation is necessary to obtain high quantum yields in core InP QDs.

### Overcoming the Limitations of the HF Treatment

The in situ HF treatment offers a safer and easy alternative to standard HF treatments on InP QDs. While it strongly enhances the PLQY, it also results in a loss of InP material due to etching and causes broadening of the optical absorption and emission, which would ideally be prevented by optimizing the reaction conditions. However, the gaseous nature of HF poses difficulties in controlling the kinetics and in the scale-up of this high-temperature treatment. In fact, according to our in situ HF experiments, a high concentration of HF (∼1000 molecules of benzoyl fluoride per QD) is required to obtain QDs with high PLQYs, which comes at the expense of extensive etching. Therefore, the ability to regulate the activity of HF at such high temperatures could be highly desirable. We investigated whether this could be achieved by introducing the base triethylamine in our in situ HF treatment, as it can bind to HF in a simple acid–base equilibrium and is inert toward benzoyl fluoride and therefore does not interfere with the amidation process. As shown in Figure S16, we find that the introduction of triethylamine allows to mitigate the loss of material while still achieving the same increase in PLQY. We expect the slight excess of octylamine in our experiments to also decrease the strength of HF in a similar fashion.

As shown in previous sections, z-type passivation plays a key role in PLQY improvement. It was also previously shown that treatments with z-type ligands such as CdCl_2_^48^ and both cadmium and zinc carboxylates^45^ can increase the PLQY of InP QDs. Therefore, we explored whether providing additional z-type ligands during the HF treatment would further improve the PLQY of the InP QDs. We observe that running the in situ HF treatment in the presence of additional InF_3_ powder does not lead to improved PLQYs. InF_3_ does not dissolve well in common solvents even when additional L-type ligands like amines or phosphines are added, potentially making the passivation of the surface by additional InF_3_ more difficult. In contrast, Zn salts like ZnCl_2_ can be dissolved with L-type ligands.^48^ We dissolved ZnCl_2_ with tributylphosphine (TBP) in mesitylene and added this to QD dispersion before performing the in situ HF treatment as described above. This results in an increase of the PLQYs up to 85%, as shown in Figure 1. This PLQY value is significantly higher than the previous record for core-only InP QDs (54%^45^) and comes close to the most efficient green-emitting InP-based core/shell structures^31^ and core-only II–IV materials.^53,54^

The results presented in this paper demonstrate that highly efficient narrow-band InP QDs can be fabricated through appropriate surface engineering, without the need to grow inorganic shells. These results provide insight into the mechanisms of carrier trapping at the surface of InP QDs but may also be relevant for the application of core-only InP QDs. For instance, the use of wide band gap shells reduces the blue absorption in films of core/shell InP QDs, compared to films of core-only InP QDs of the same thickness. The brightness of a film of QDs depends on their PLQY as well as their absorptance and is thus higher for the core-only InP QDs than for core–shell QDs. In addition, charge transport is more efficient in core-only QD films because inorganic shells introduce additional tunnel barriers for electrons and holes. Efficient charge transport is important for applications such as electroluminescent QLEDs, photodetectors, and solar cells. Our work shows that core-only (fluoride-terminated) InP QDs could be a Pb- or Cd-free alternative for use in these applications, although their stability remains to be investigated.

## Conclusion

Using a safe, water-free in situ HF treatment, the quantum yield of InP quantum dots can be increased from <0.1% to up to 70% and up to 85% when additional z-type ZnCl_2_ ligands are provided. Optical analyses show that in situ generated HF etches InP QDs to produce InF_3_ and PH_3_, resulting in a loss of material and a minor blue-shift of the optical properties. Structural analyses show that the in situ HF treatment does not remove all oxidized phosphorus from the QD surface but converts polyphosphate species to H_*x*_PO_4_ and results in exchange of surface In(PA)_3_ for InF_3_. On the basis of these results in combination with DFT calculations, phosphorus dangling bonds and the presence of polyphosphates on the surface are suggested to be the sources of traps that cause nonradiative recombination in InP QDs. Removing these traps results in core-only InP QDs with PLQY that is comparable to the best InP/ZnSe/ZnS core/shell/shell QDs.

## Supporting Information Available

Computational details, additional optical, ssNMR, solution NMR and XPS data, additional DFT calculations (PDF)
