# Supplementary material for: A Water-Free In Situ HF Treatment for Ultrabright InP Quantum Dots
Source: Chem Mater. 2022 Nov 4;34(22):10093–103. doi: 10.1021/acs.chemmater.2c02800 (PMC9686131; doi:10.1021/acs.chemmater.2c02800)
Supplement: Supplementary file 1 — cm2c02800_si_001.pdf [file cm2c02800_si_001.pdf]

SUPPORTING INFORMATION FOR

A water-free in-situ HF treatment for ultra-bright InP quantum dots

*Reinout F. Ubbink[†], Guilherme Almeida[†], Hodayfa Iziyi[†], Indy du Fossé[†], Ruud Verkleij[†], Swapna Ganapathy[‡], Ernst R. H. van Eck[§] and Arjan J. Houtepen[†]**

[†]Optoelectronic Materials Section, Faculty of Applied Sciences, Delft University of Technology, Van der Maasweg 9, 2629 HZ Delft, The Netherlands

[‡] Department of Radiation Science and Technology, Faculty of Applied Sciences, Delft University of Technology, 2629JB Delft, The Netherlands

[§] Magnetic Resonance Research Center, Institute for Molecules and Materials, Radboud University, 6525 AJ Nijmegen, The Netherlands

Computational methods

Geometry optimization of complexes for NMR were carried out using a PBE exchange correlation functional[1] and a TZ2P basis set. The structure of H_3PO_4, which is used as the chemical shift reference, was taken directly from the ADF database. Scalar relativistic effects were taken into account by the zeroth-order regular approximation (ZORA). All NMR calculations[2,3] were carried out using a hybrid PBE0 exchange correlation functional[7] and TZ2P-J basis set with the inclusion of spin-orbit coupling as implemented in the ADF software package.[4] Calculations on QD systems were carried out at the DFT level, using a PBE exchange-correlation functional[1] and double-ζ basis set, as implemented in the CP2K quantum chemistry software package.[5] Relativistic

effects were taken into account by using effective core potentials. Geometry optimizations were performed at 0 K and in the gas phase.

To confirm the orbital localization of the trap state, the inverse participation ratio (IPR)[6,7] of an electronic state is used. We define IPR as:

$$IPR_i = \frac{\sum_a |P_{a,i}|^4}{\left(\sum_a |P_{a,i}|^2\right)^2}$$

Where $P_{a,i}$ is the weight of a molecular orbital i on a given atom a expanded in an atomic orbital basis. The IPR can be used to estimate the number of atoms that contribute to an electronic state. The value can range from $\frac{1}{\text{\# atoms present in the system}}$, meaning the contribution is equally distributed over all atoms, to 1, meaning that the contribution comes from 1 atom.

Crystal-orbital overlap population (COOP) is used to give insight on the bonding or anti-bonding nature of an electronic state.[8–10] Positive COOP values correspond to a bonding interaction, whereas negative COOP values correspond to an anti-bonding interaction. A value close to 0 is a non-bonding interaction.

IPR & COOP calculations were carried out using the QMFlows Python package.[11]

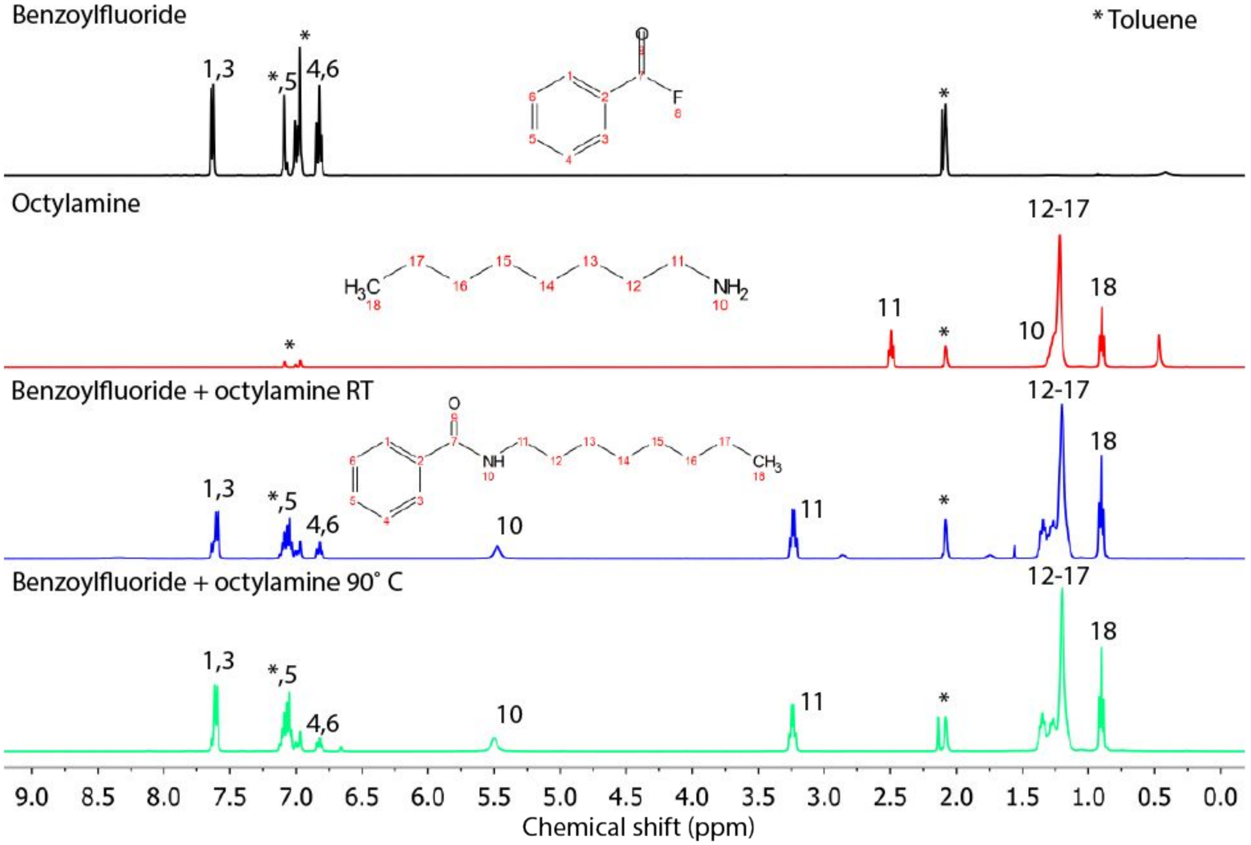

Figure S1. Solution 1H spectra of the compounds used in the in-situ HF treatment, before and after their reaction. Spectra were referenced according to the residual solvent peaks of toluene-d8 (2.08 ppm). As can be observed from the shift in peak 11 (no residual peak at 2.5 ppm after the reaction), full conversion of the amine is achieved when equimolar amounts are reacted.

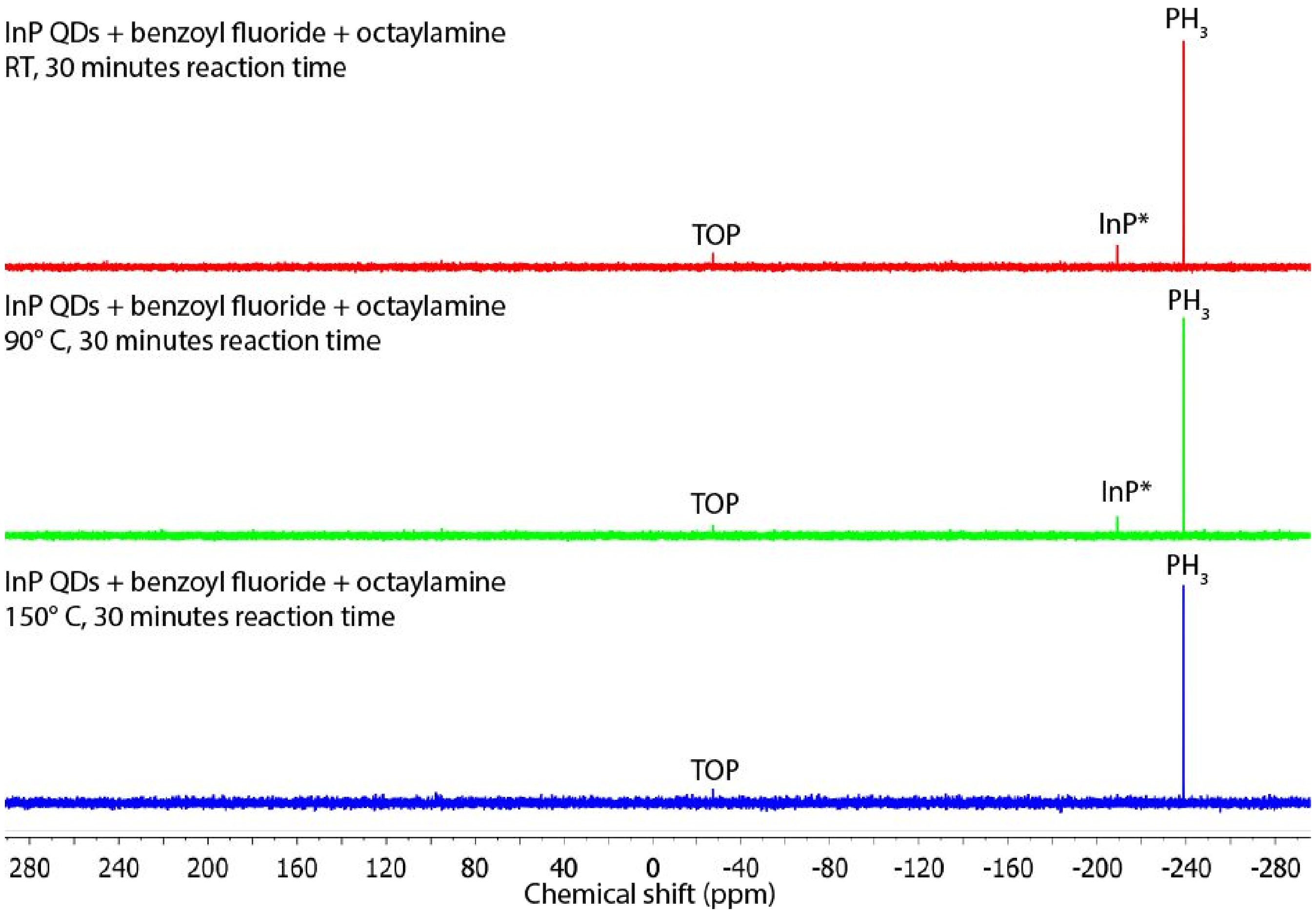

Figure S2. Solution ^{31}P NMR spectra of aliquots taken from treatments at different temperatures. The PH_3 peak can clearly be observed at -239 ppm. Free TOP is also present in all spectra at -29 ppm. At -210 ppm, a final peak can be observed, but only in the RT and 90 ˚C spectra. This peak shows at the typical shift of InP (see solid state NMR spectra in Figure S10), and is thus ascribed to small InP clusters/complexes in solution.

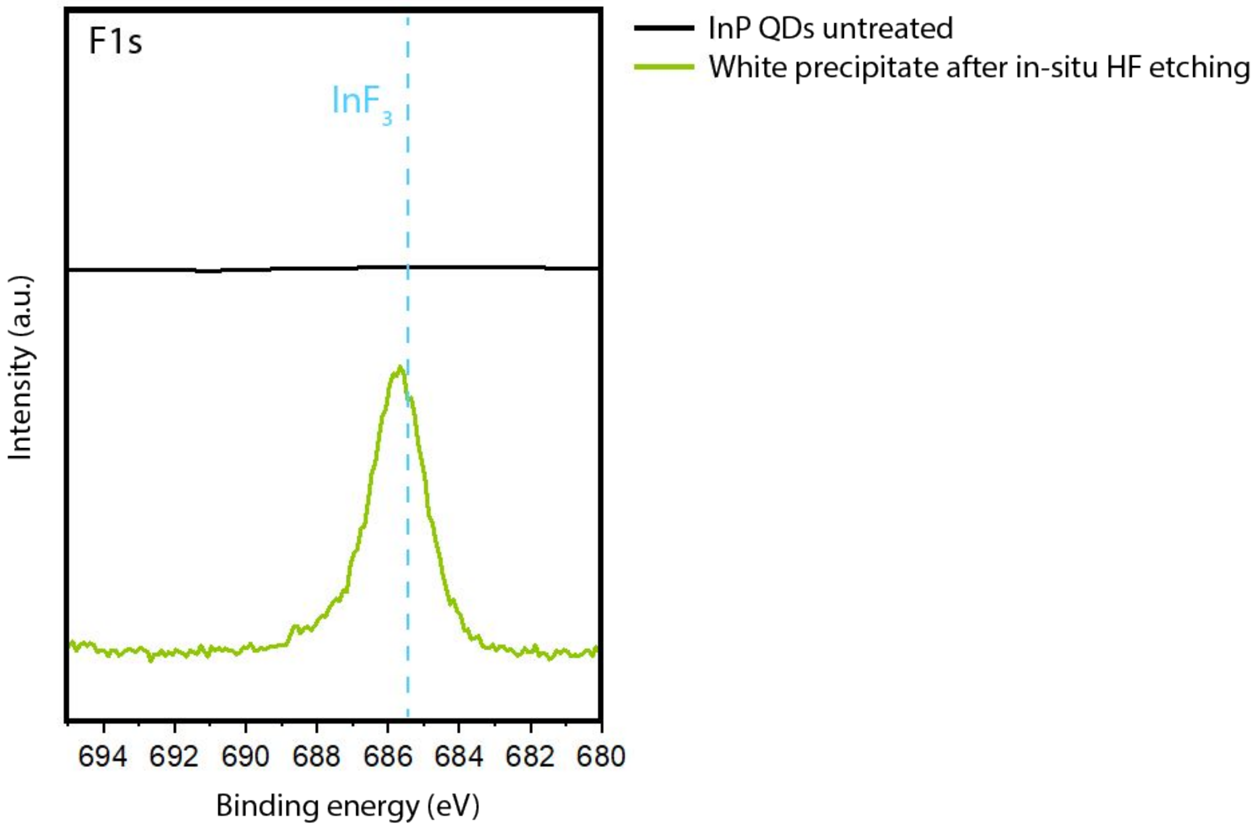

Figure S3. F1s XPS spectra of the as-synthesized QDs and the white powder that formed after reacting an excess of in-situ generated HF with InP QDs. All of the InP has been converted to InF_3 during the treatment.

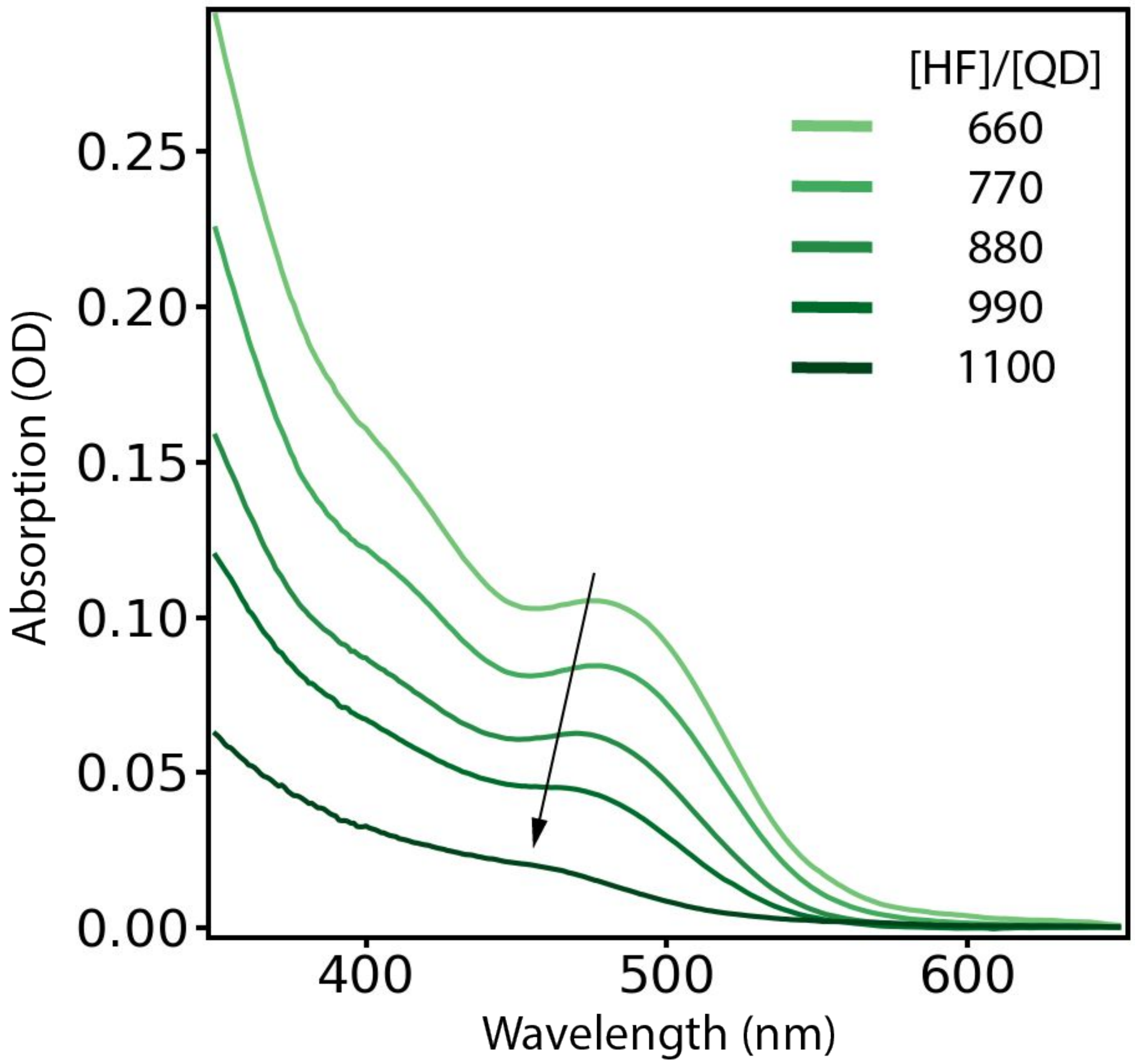

Figure S4. Absolute absorption spectra of InP QDs after in-situ HF etching at different concentrations. HF concentration was varied by adjusting both the amount of benzoyl fluoride and octylamine that was added while keeping their ratio consistent. At higher HF concentrations, the first absorption maximum is blueshifted further and a larger drop in the absorption is observed.

The highest quantum yields were observed for HF concentrations between 850-1000 molar equivalents.

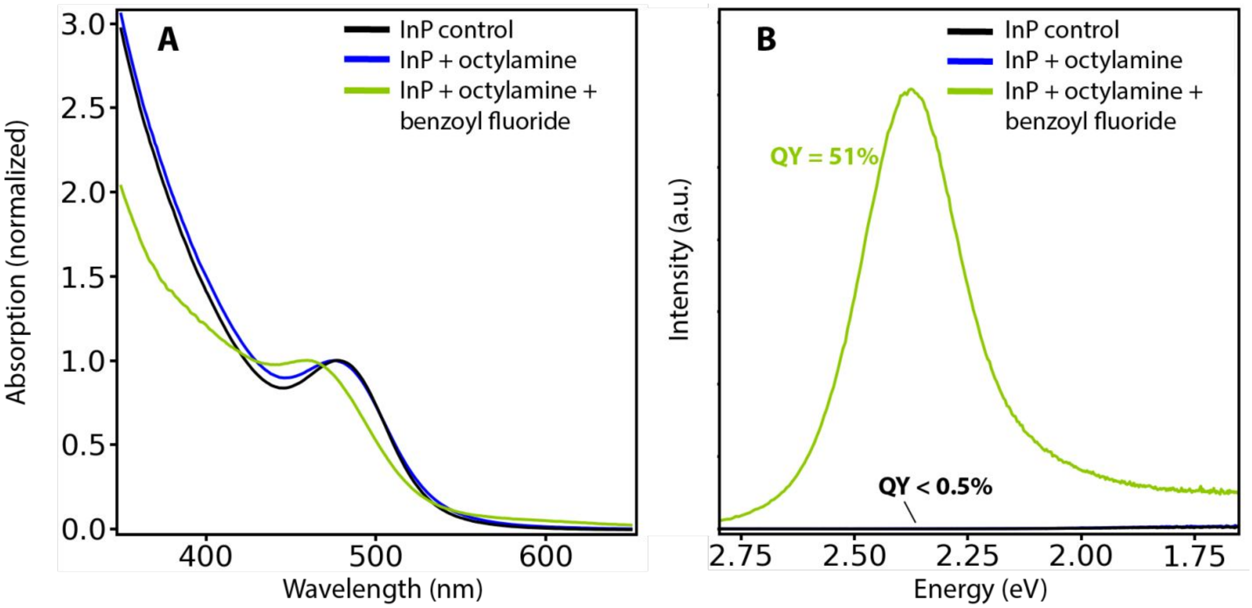

Figure S5. Normalized absorption (A) and PL emission (B) spectra of InP treated with octylamine and a combination of octylamine and benzoyl fluoride. [benzoyl fluoride]/[QD] = 1400, [octylamine]/[QD] = 2000. Treatments were performed at 150˚C for 180 minutes in mestitylene, similar to the in-situ HF treatment.

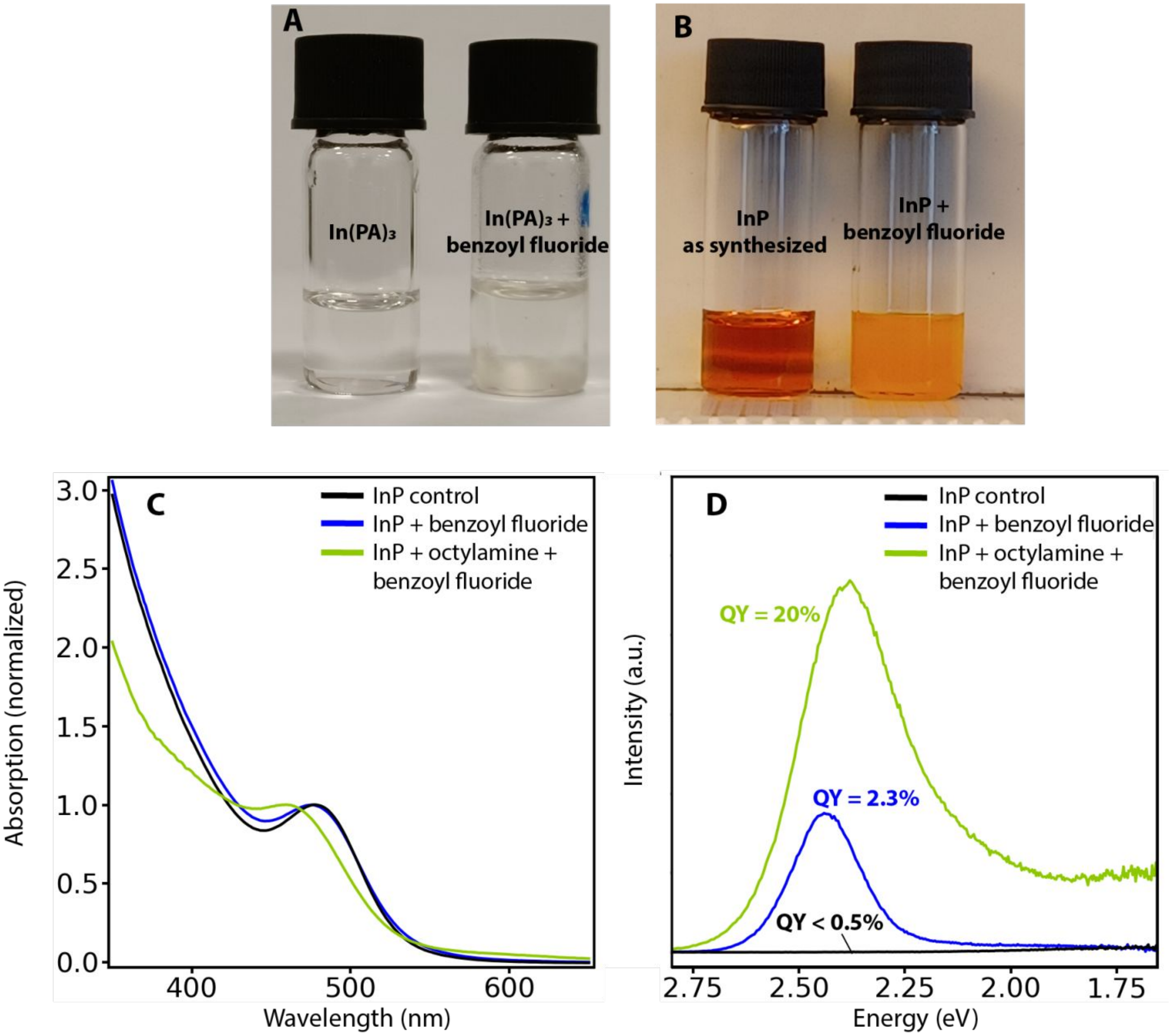

Figure S6. Photographs and optical data of InP QDs treated with benzoyl fluoride. Photographs of (A) $In(Pa)_3$ and (B) InP QDs in toluene before and after reaction with benzoyl fluoride. A precipitate is formed during the reaction of benzoyl fluoride and indium palmitate. The InP QDs have precipitated after the reaction with benzoyl fluoride, indicating a loss of polar surface ligands. Normalized absorption (C) and PL emission (D) spectra of InP treated with benzoyl fluoride and

a combination of octylamine and benzoyl fluoride (in-situ HF). [benzoyl fluoride]/[QD] = 1400, [octylamine]/[QD] = 2000. The benzoyl fluoride was performed for 10 minutes at 150 ˚C, while the in-situ HF treatment was performed for 5 minutes at 150 ˚C.

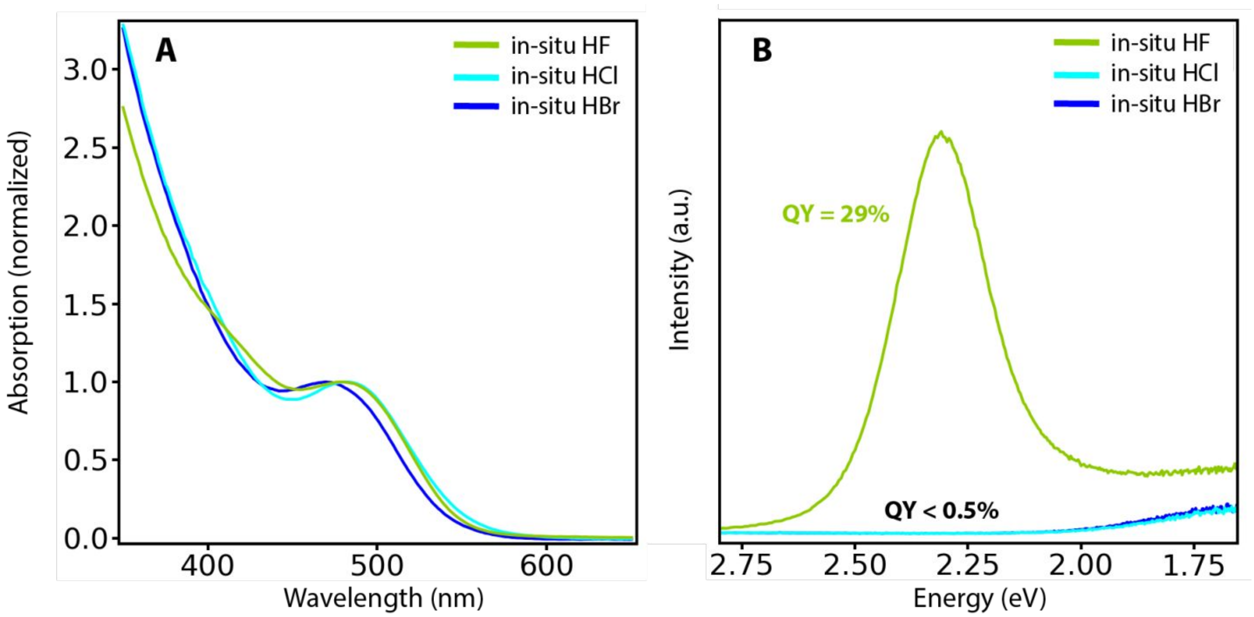

Figure S7. Normalized absorption (A) and PL emission (B) spectra of InP treated with different in-situ generated acids. Benzoyl bromide, benzoyl chloride and benzoyl fluoride were used in conjunction with octylamine to produce each of the acids in-situ. [acyl halogen]/[QD] = 700 for each of the samples. At higher concentrations, HCl and HBr fully dissolved the InP. Treatments were performed at 150˚C for 180 minutes in mesitylene, similar to the in-situ HF treatment.

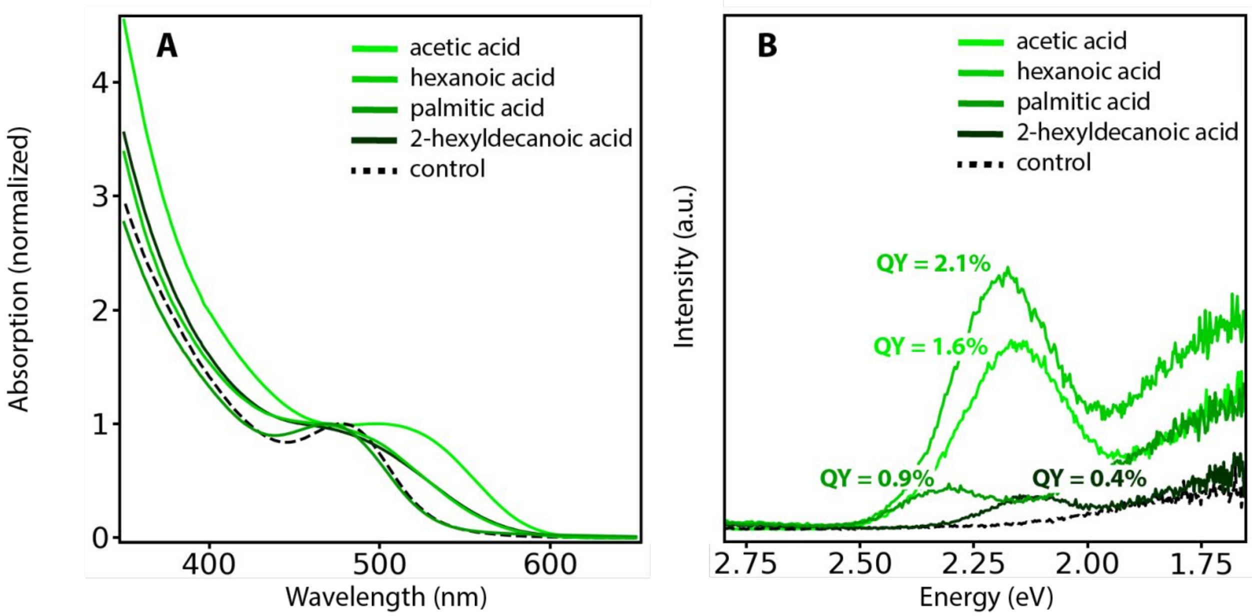

Figure S8. Normalized absorption (A) and PL emission (B) spectra of InP treated with different organic acids. [acid]/[QD] = 1000 for each of the samples. Treatments were performed at 150˚C for 180 minutes in mesitylene, similar to the in-situ HF treatment. Modest increases in quantum yield are observed when smaller organic acids are applied.

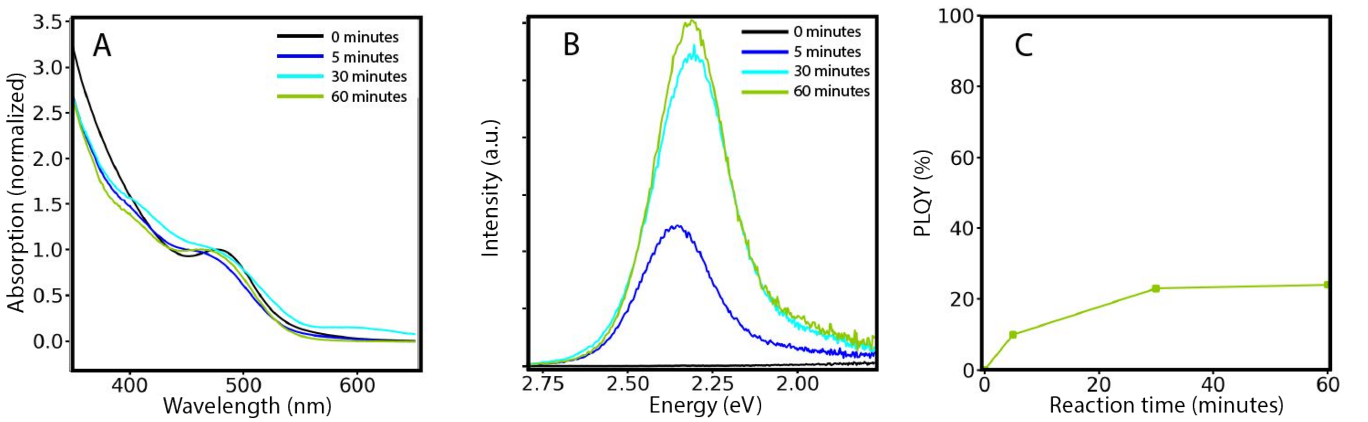

Figure S9. Optical data of the InF3 treatment at 150 ˚C. (A) normalized absorption curves. (B) PL spectra. (C) photoluminescence quantum yield over time during the treatment. The treatment is performed by adding solid InF_3 to a dilute solution of InP QDs in mesitylene and heating to 150 ˚C. No additional compounds need to be added.

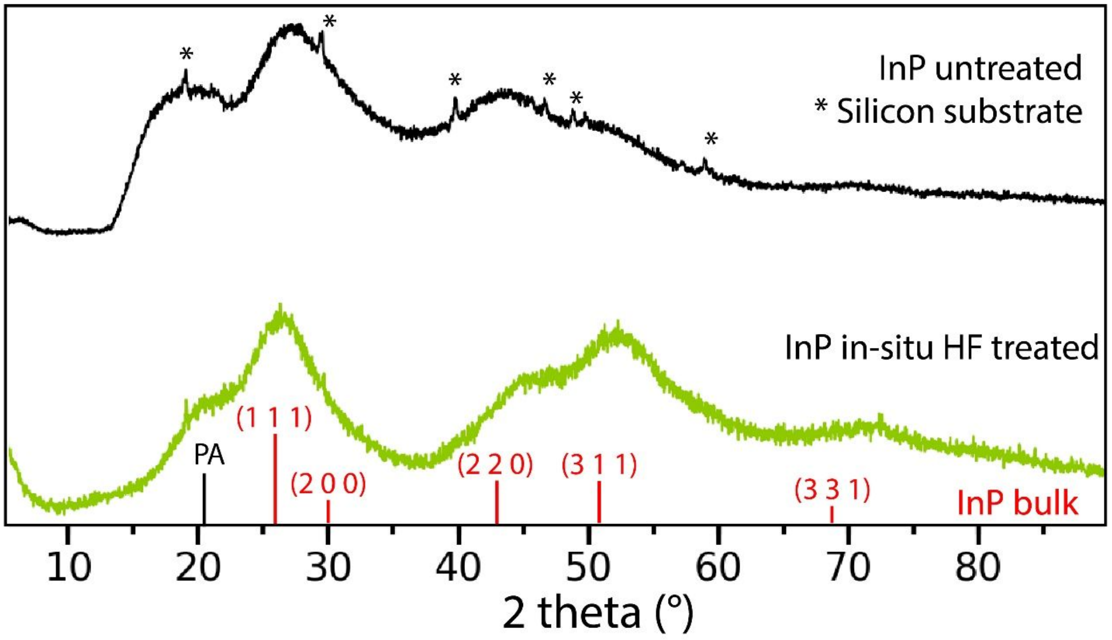

Figure S10. X-ray diffraction patterns of InP QDs before and after the in-situ HF treatment. After the treatment the peak at 20°, ascribed to ordered palmitate ligands, is decreased in intensity.

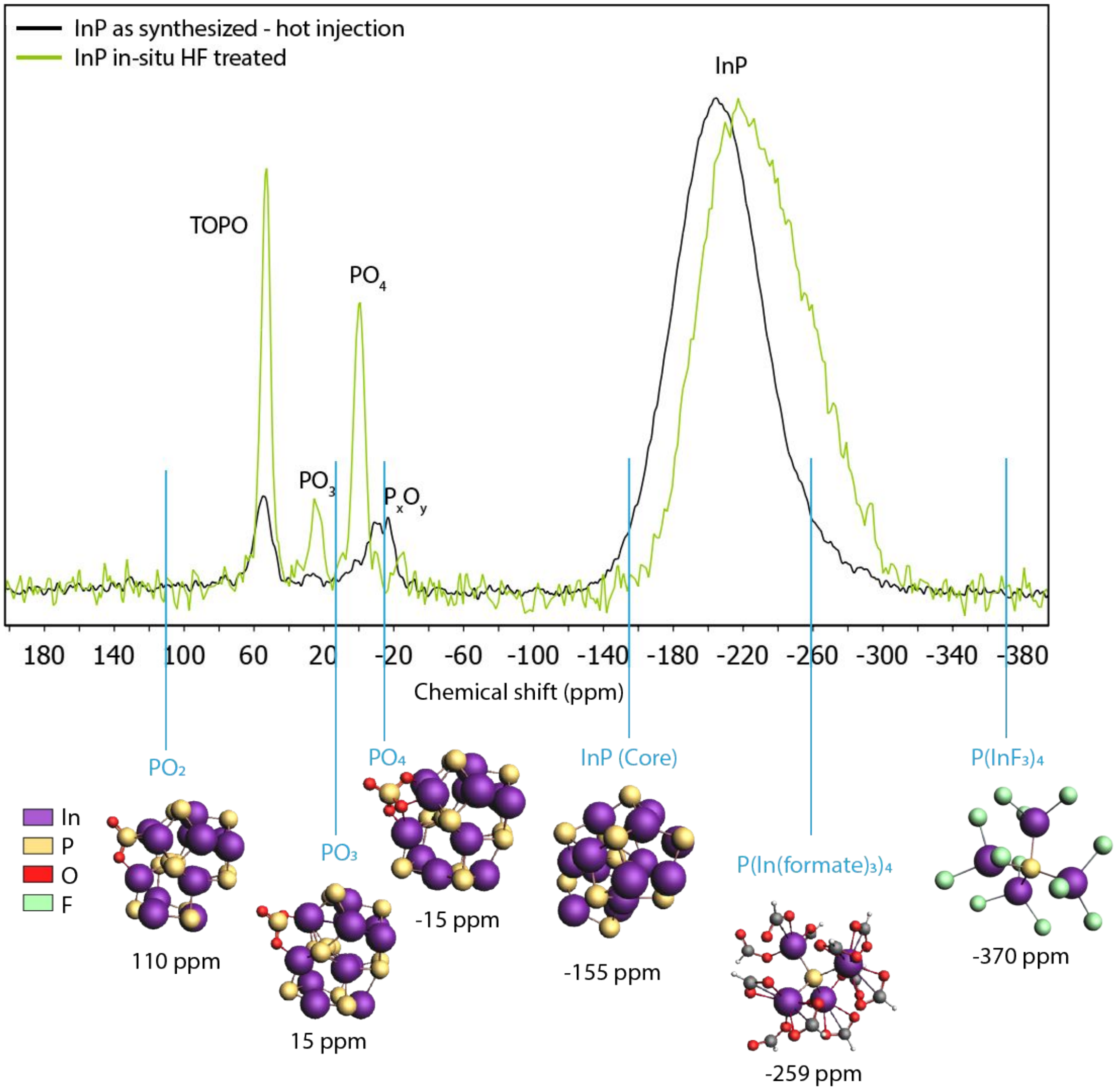

Figure S11. ^{31}P NMR shifts of various small InP structures as calculated using DFT. Shifts were referenced using the calculated shift of H_3PO_4. For PO_x species, lower chemical shift is observed for increasing x, which mirrors experimental observations. P bound to four InF_3 was calculated to have a lower chemical shift than P bound to four $In(carbonate)_3$. This is in accordance with our observations after the HF treatment, were the ^{31}P chemical shift becomes more negative after $In(PA)_3$ has been replaced with InF_3 on the InP surface.

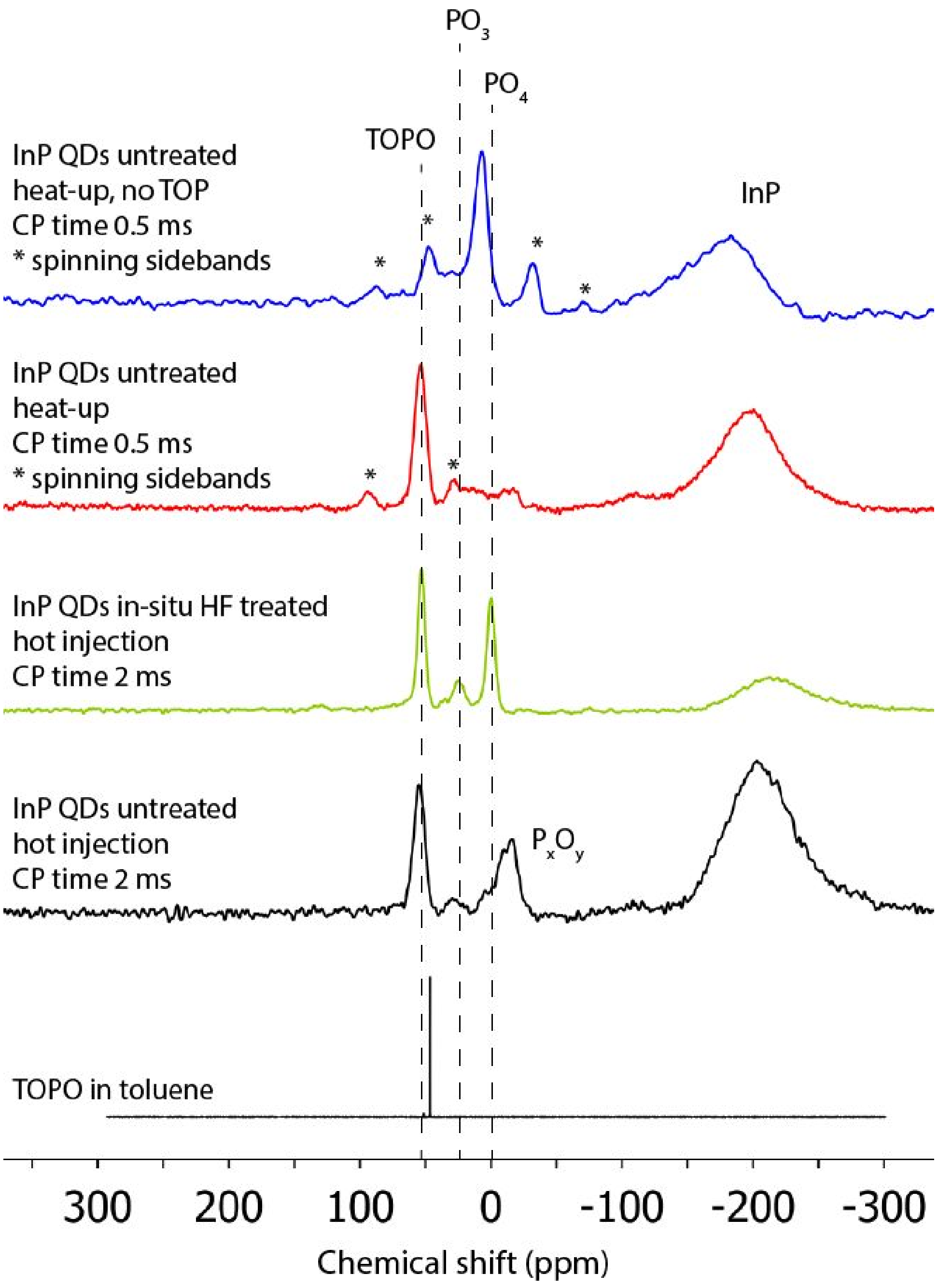

Figure S12. $^{1}H \rightarrow ^{31}P$ CPMAS spectra of as-synthesized QDs from heat-up syntheses and QDs from the hot injection synthesis before and after the in-situ HF treatment. The surface peaks in the region of 100 to -20 ppm are enhanced compared to the InP core peak due to proximity to hydrogen

atoms. Dashed lines indicate the chemical shifts of PO_4, PO_3 and TOPO species. TOPO in toluene is provided for reference.

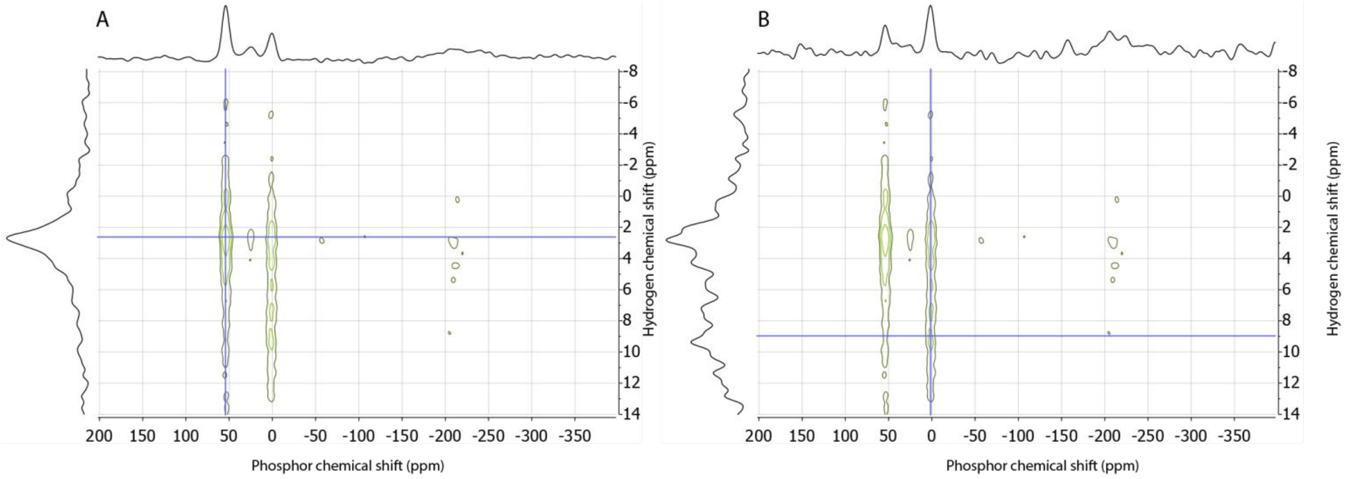

Figure S13. Projections of the $^{31}P\{^1H\}$ HETCOR spectra after the in-situ HF treatment. The phosphorus slice around ^1H 2.5 ppm shows spatial proximity of the aliphatic protons to the phosphorus peak at 52 ppm. The slice at ^1H 9 ppm shows a correlation between the PO_4 species at 0 ppm and the new, broad, downfield hydrogen peak around 7.5 ppm.

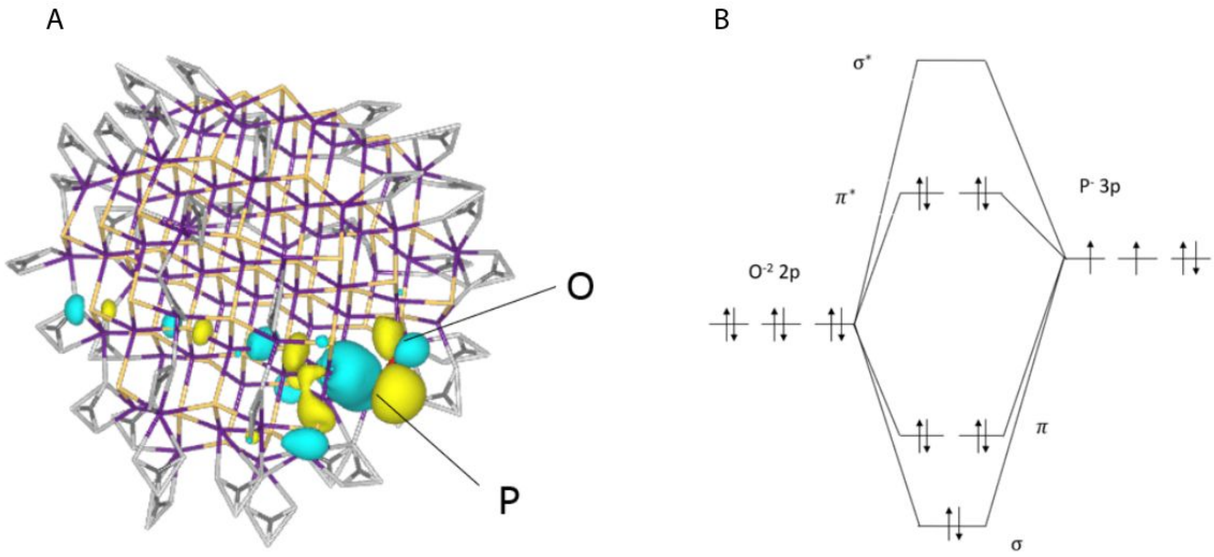

Figure S14. (A) Contour plot of the HOMO in the PO_2^{3-} InP QDs DFT simulations. (B) Molecular orbital (MO) of PO^{3-} complex. In the DOS InP QDs containing PO_2^{3-}, a state just above the valence band (VB) is formed, with significant contribution from the added oxygen atom as well as the phosphor atom the oxygen is bound to (see Figure 4). The IPR value of the trap state of the PO moiety is 0.33, whereas the IPR value of the unoxidized HOMO is 0.1 which shows that the trap state is slightly localized. The contour plot of this localized wave function (A) indicates that it is formed by an anti-bonding combination of p orbitals on the surface P and O atoms. To confirm the anti-bonding nature of the trap state, we use the crystal-orbital overlap population (COOP) analysis. Positive COOP values correspond to a bonding interaction, whereas negative COOP values correspond to an anti-bonding interaction. A value around 0 is a non-bonding interaction. The negative COOP values confirm the anti-bonding nature of the state (see Figure 4). Therefore we conjecture that the state inside the bandgap is primarily made up from the anti-bonding orbital of the O 2p orbital and the P 3p orbital. To further confirm this, we set up the molecular orbital (MO) diagram of PO^{3-} (B) We see that the highest occupied molecular orbital (HOMO) is indeed a π^* orbital.

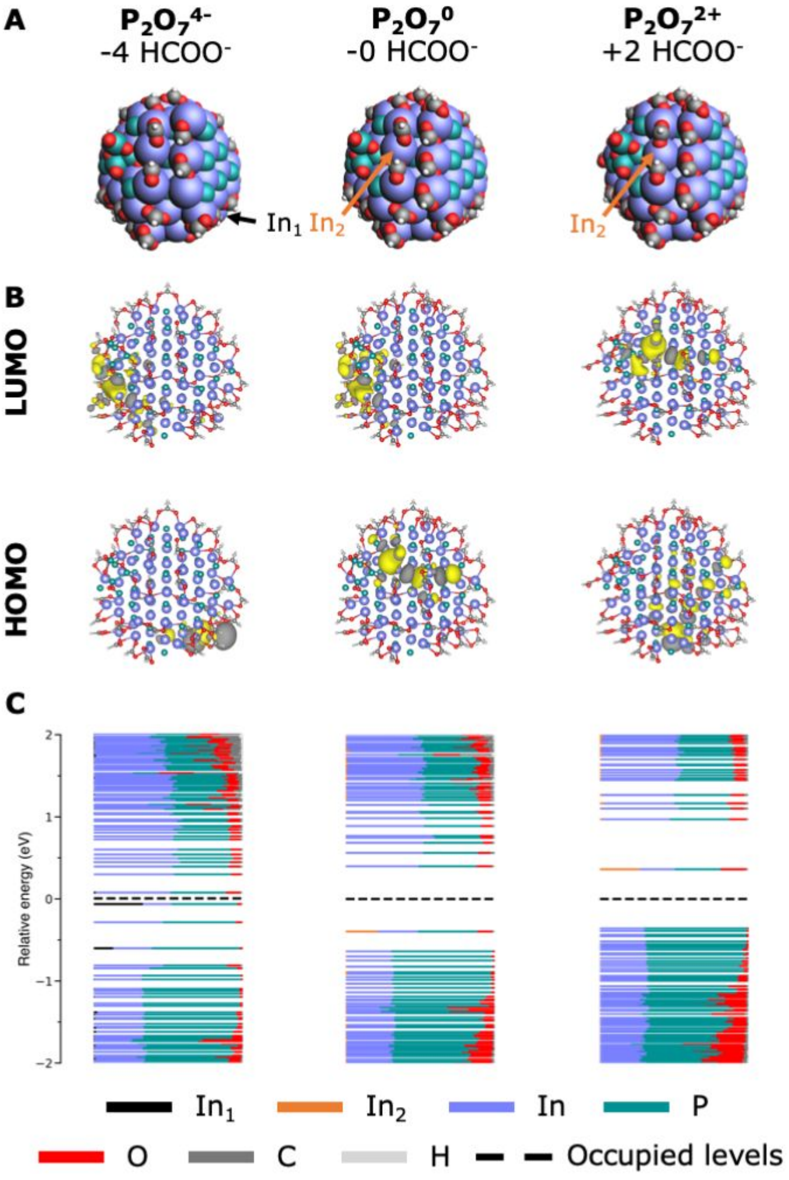

Figure S15. DFT calculations on InP QDs containing a P_2O_7 species on the surface. (A) atomic structures after geometry optimization. (B) HOMO and LUMO orbital shapes. (C) DOS plots of the respective structures. Negatively charged and neutral P_2O_7 species result in n-doping of the QD and reduction of indium, similar to earlier results on CdX QDs. Achieving charge neutrality is only possible by adding two additional carbonate ligands, effectively resulting in an unphysical $P_2O_7^{2+}$ species. Furthermore P_2O_7 species are not stable in DFT calculations, resulting in spontaneous

surface reconstructions, where (O_2P)-O-(PO_3) units are formed, and one oxygen forms an In-O-P bond instead.

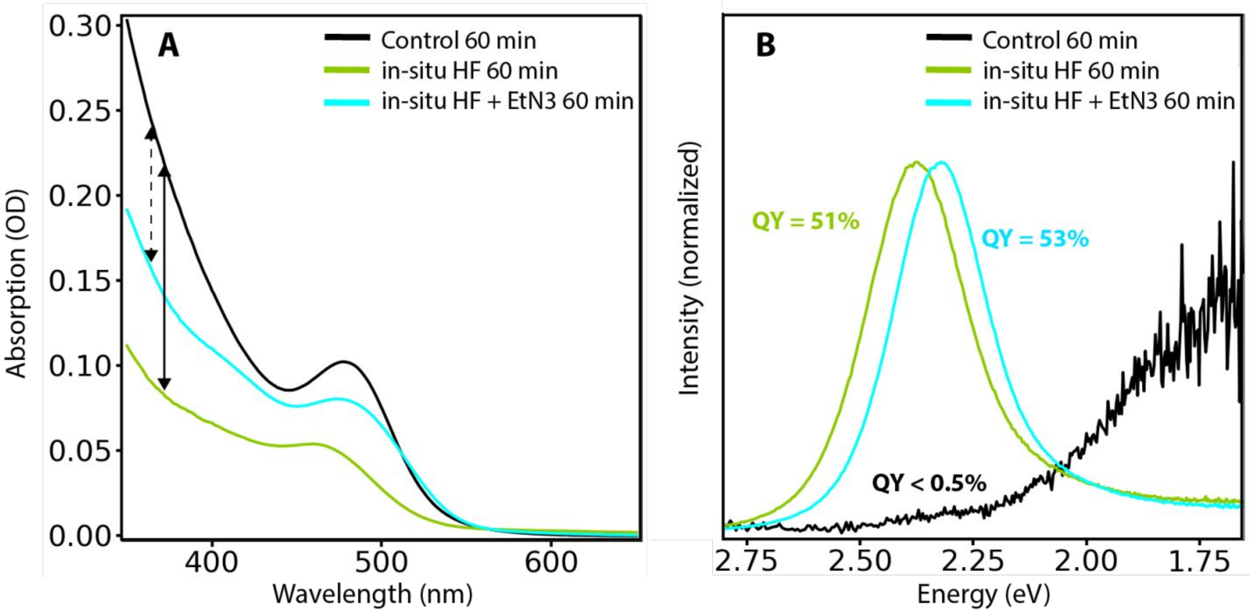

Figure S16. Optical data of the in-situ HF treatment and the in-situ HF treatment with added triethylamine at 150 ˚C, compared to a control sample at 150 ˚C. Triethylamine was added at the same concentration as the benzoyl fluoride. (A) absolute absorption spectra. (B) PL spectra. Adding triethylamine before starting the treatment results in less HF etching, while achieving the same increase in PLQY. Less InP is converted to InF_3 as can be seen by the reduced absorption

drop compared to the HF treatment. There is also no absorption or PL blueshift observed after the treatment.

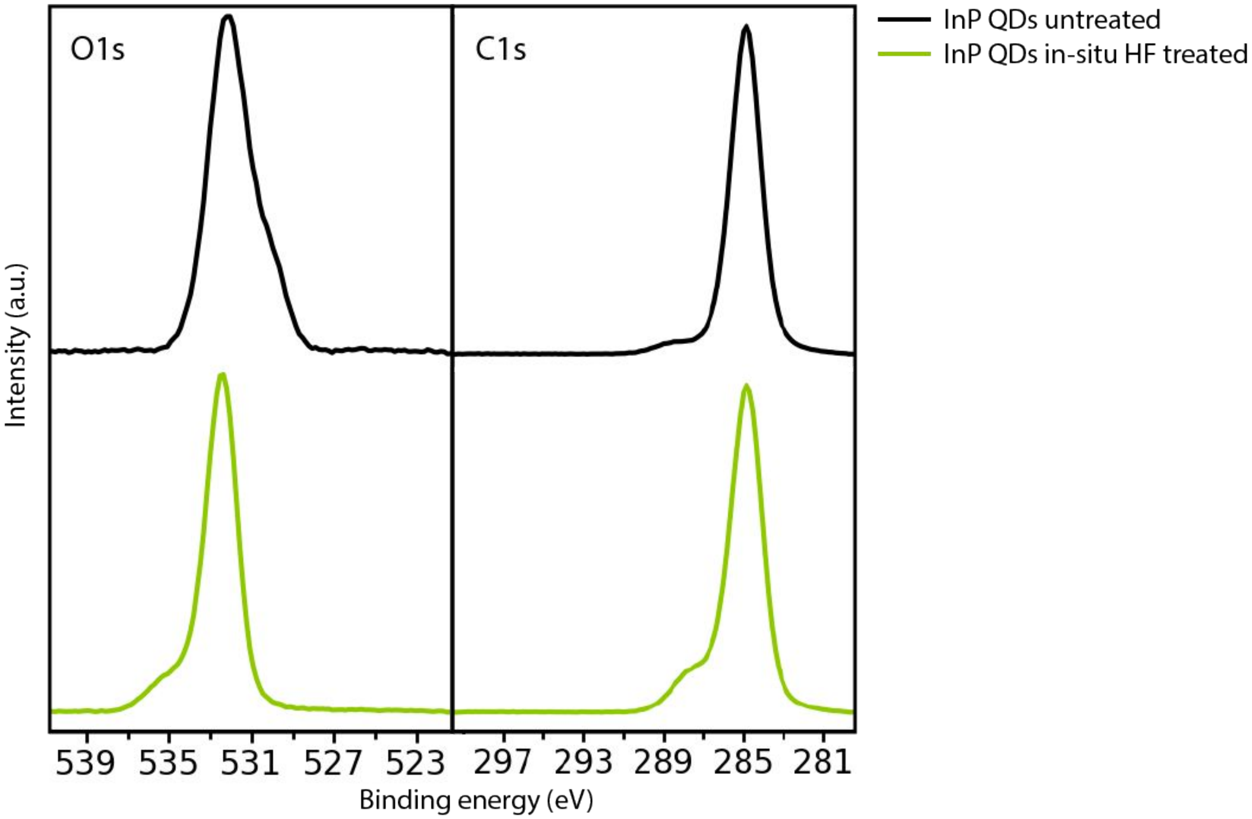

Figure S17. O1s and C1s XPS spectra of the InP QDs before and after the in-situ HF treatment. It should be noted that after the in-situ HF treatment, less carbonate ligands are present on the InP surface than before the treatment. The dropcasting procedure of the samples after the treatment also resulted in thinner QD layers with a lower coverage of the substrate. Because of these factors,

the signal from contaminations (organic compounds on the substrate) in the O1s and C1s spectra is expected to be higher in the in-situ HF treated samples relative to the untreated samples, which makes a comparison of these spectra difficult.

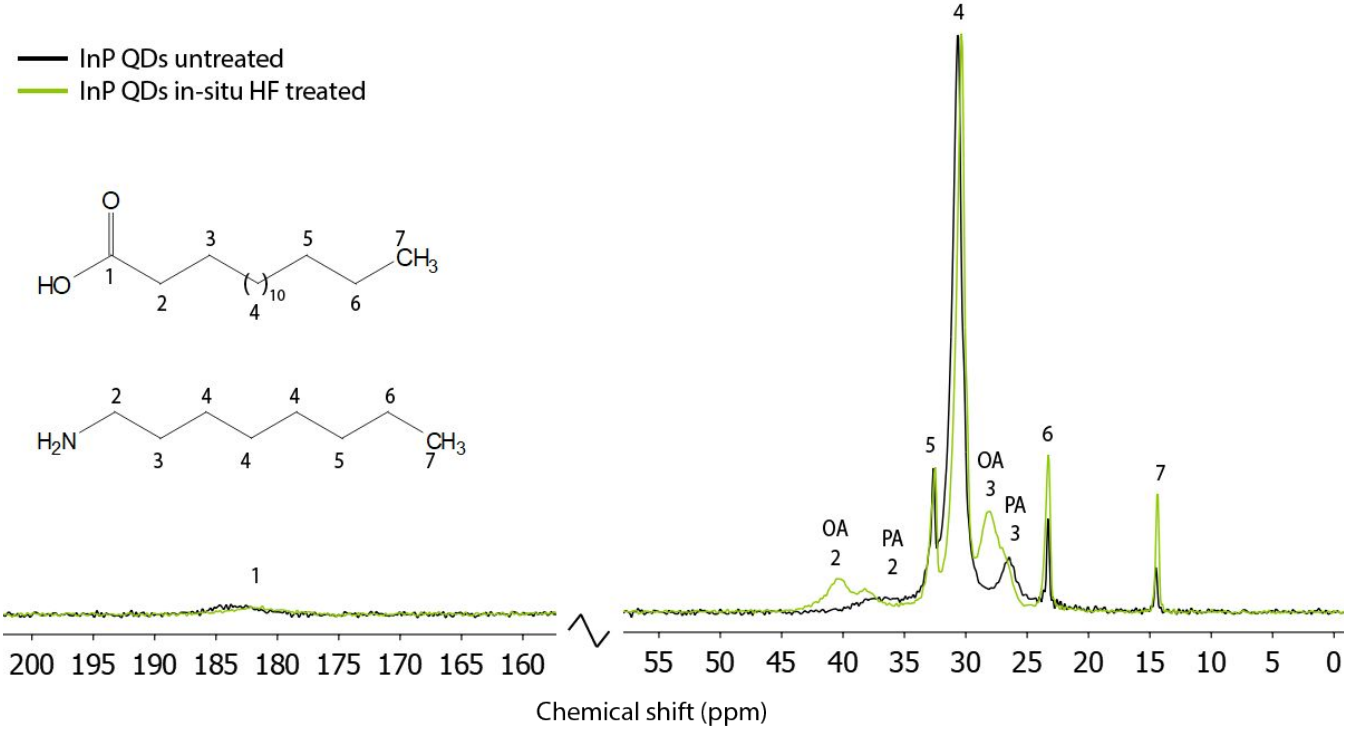

Figure S18. ^{13}C ssNMR spectra. After the in-situ HF treatment, octylamine is bound to the surface in addition to some remaining palmitates.

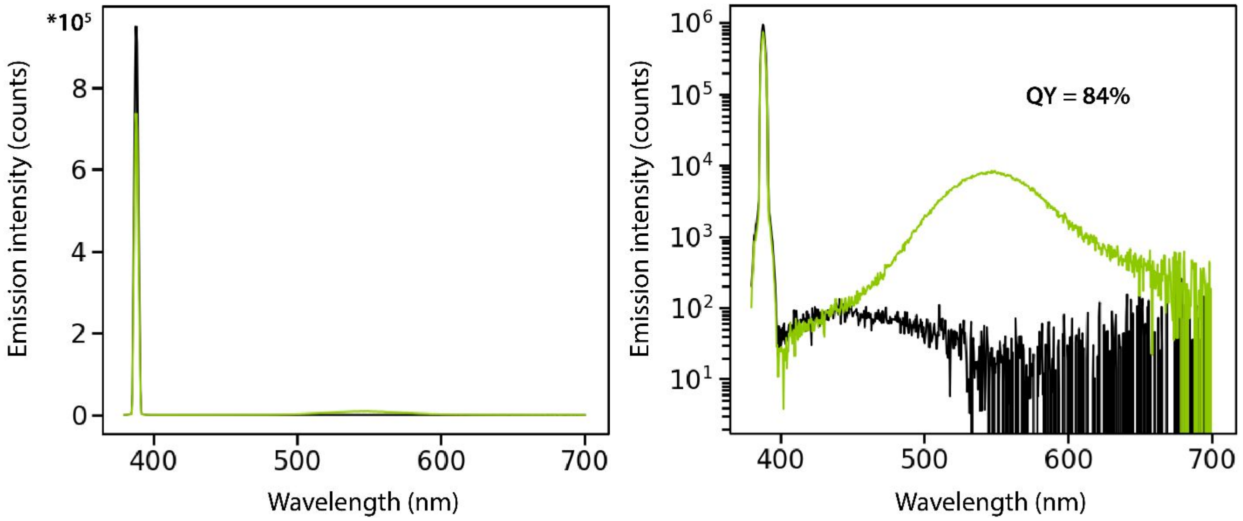

Figure S19. Integrating sphere PLQY measurement of a typical in-situ HF/$ZnCl_2$ treated InP QD sample. A PLQY of 84% was observed.

REFERENCES FOR SUPPORTING INFORMATION

1. Perdew, J. P.; Burke, K.; Ernzerhof, M. Generalized Gradient Approximation Made Simple. *Phys. Rev. Lett.* **1996**, *77* (18), 3865–3868.

2. Krykunov, M.; Ziegler, T.; Lenthe, E. van. Hybrid Density Functional Calculations of Nuclear Magnetic Shieldings Using Slater-Type Orbitals and the Zeroth-Order Regular Approximation. *Int. J. Quantum Chem.* **2009**, *109* (8), 1676–1683.

3. Schreckenbach, G.; Ziegler, T. Calculation of NMR Shielding Tensors Using Gauge-Including Atomic Orbitals and Modern Density Functional Theory. *J. Phys. Chem.* **1995**, *99* (2), 606–611.

4. te Velde, G.; Bickelhaupt, F. M.; Baerends, E. J.; Fonseca Guerra, C.; van Gisbergen, S. J. A.; Snijders, J. G.; Ziegler, T. Chemistry with ADF. *J. Comput. Chem.* **2001**, *22* (9), 931–967.

5. Hutter, J.; Iannuzzi, M.; Schiffmann, F.; VandeVondele, J. Cp2k: Atomistic Simulations of Condensed Matter Systems. *Wiley Interdiscip. Rev. Comput. Mol. Sci.* **2014**, *4* (1), 15–25.

6. Murphy, N. C.; Wortis, R.; Atkinson, W. A. Generalized Inverse Participation Ratio as a Possible Measure of Localization for Interacting Systems. *Phys. Rev. B - Condens. Matter Mater. Phys.* **2011**, *83* (18), 27–29.

7. Dümbgen, K. C.; Zito, J.; Infante, I.; Hens, Z. Shape, Electronic Structure, and Trap States in Indium Phosphide Quantum Dots. *Chem. Mater.* **2021**, *33* (17), 6885–6896.

8. Maintz, S.; Deringer, V. L.; Tchougréeff, A. L.; Dronskowski, R. LOBSTER: A Tool to Extract Chemical Bonding from Plane-Wave Based DFT. *J. Comput. Chem.* **2016**, *37* (11), 1030–1035.

9. Diaz-Lopez, M.; Guda, S. A.; Joly, Y. Crystal Orbital Overlap Population and X-Ray Absorption Spectroscopy. *J. Phys. Chem. A* **2020**, *124* (29), 6111–6118.

10. Dronskowski, R.; Blöchl, P. E. Crystal Orbital Hamilton Populations (COHP). Energy-Resolved Visualization of Chemical Bonding in Solids Based on Density-Functional Calculations. *J. Phys. Chem.* **1993**, *97* (33), 8617–8624.

11. Zapata, F.; Ridder, L.; Hidding, J.; Jacob, C. R.; Infante, I.; Visscher, L. QMflows: A Tool Kit for Interoperable Parallel Workflows in Quantum Chemistry. *J. Chem. Inf. Model.* **2019**, *59* (7), 3191–3197.
